# Biomechanics of the Attachment of the Intertidal Seaweed *Fucus distichus*

**DOI:** 10.1093/iob/obag010

**Published:** 2026-03-27

**Authors:** F Klimm, A P Summers, T Speck

**Affiliations:** Plant Biomechanics Group @ Botanic Garden, University of Freiburg, D-79104 Freiburg, Germany; Cluster of Excellence livMatS @ FIT—Freiburg Center for Interactive Materials and Bioinspired Technologies, University of Freiburg, D-79110 Freiburg, Germany; Friday Harbor Laboratories, University of Washington, Friday Harbor, WA 98250, USA; Plant Biomechanics Group @ Botanic Garden, University of Freiburg, D-79104 Freiburg, Germany; Cluster of Excellence livMatS @ FIT—Freiburg Center for Interactive Materials and Bioinspired Technologies, University of Freiburg, D-79110 Freiburg, Germany

## Abstract

Benthic marine seaweeds are subjected to considerable hydrodynamic loads which may damage and dislodge them. Different forms of attachment systems assure that they remain in place, such as the attachment disc of the intertidal brown alga *Fucus distichus*. Discerning how such attachments function helps to understand the seaweed’s ecological performance, may unravel interesting mechanical design features and in future may even inspire biomimetic attachment structures. Methodologically, the marine field setting is challenging and often limits comprehensive mechanical analysis. In this study, we present a setup that allows continuous *in situ* mechanical tests. We apply it to dislodge the attachment of young individuals of *F. distichus* (disc and stipe base), cross-validate our results with tensile tests in the lab, and present a thorough mechanical characterization of the attachment structure. Even though the stipes of *F. distichus* are weak (low breaking strength) and compliant (low elastic modulus in tension), they are thick and extensible and the work required to dislodge the attachment structure is primarily required to stretch the stipes in their elastic range. Discs are weaker than stipes (lower breaking strength), probably due to weak substrate cohesion rather than weak disc adhesion. This stresses the importance of the stipe broadening into a disc to provide a wide contact area and thereby a well-balanced attachment structure. Complementary analyses of thallus morphology and size distribution suggest that for young (small) thalli, the risk of dislodgement due to drag does not dictate thallus size. However, substrate cohesion at the sampled site may impose upper mechanical constraints to thallus size. *Fucus distichus* thalli not only resist drag with their attachment, but we illustrate in flume experiments that in water flow, thalli also bent down toward the substrate and streamline considerably, which will reduce the drag the attachment has to bear.

## German abstract

Benthische Meeresalgen sind erheblichen hydrodynamischen Belastungen ausgesetzt, die sie beschädigen und von ihrem Untergrund abreißen können. Verschiedene Befestigungsformen stellen sicher, dass die Algen dauerhaft verankert bleiben, wie beispielsweise die Haftscheibe von *F. distichus*, einer Braunalge welche im Gezeitenbereich vorkommt. Die Funktionsweise solcher Haftsysteme zu verstehen hilft, die ökologische Leistungsfähigkeit der Algen nachzuvollziehen, mechanische Designmerkmale zu identifizieren und in Zukunft sogar bionische Haftsysteme zu inspirieren. Methodisch ist die marine Feldforschung eine Herausforderung, und schränkt eine umfassende mechanische Analyse oft ein. In dieser Studie stellen wir einen Aufbau vor, der kontinuierliche mechanische Tests vor Ort ermöglicht. Wir nutzen den Aufbau, um Abzugtests am Haftsystem (Haftscheibe und unteres Ende des Stiels) junger Exemplare von *F. distichus* durchzuführen, validieren unsere Ergebnisse durch Zugversuche im Labor und präsentieren eine gründliche mechanische Charakterisierung des Haftsystems. Obwohl die Stiele von *F. distichus* schwach (geringe Bruchfestigkeit) und nachgiebig (geringer Zugelastizitätsmodul) sind, sind sie dick und dehnbar, und die zum Ablösen der Befestigung erforderliche Arbeit besteht in erster Linie darin, die Stiele in ihrem elastischen Bereich zu dehnen. Die Haftscheiben sind schwächer als die Stiele (geringere Bruchfestigkeit), was wahrscheinlich eher auf schwache Substratkohäsion als auf schwache Adhäsion der Haftscheiben zurückzuführen ist. Dies unterstreicht wie wichtig es ist, dass der Stiel sich am unteren Ende zu einer Scheibe verbreitert, um eine große Kontaktfläche und damit ein in sich gut abgestimmtes Haftsystem zu gewährleisten. Ergänzende Analysen der Thallusmorphologie und Thallusgrößenverteilung legen nahe, dass für junge (kleine) Thalli das Risiko einer Ablösung durch die Strömung nicht die Thallusgröße vorgibt. Allerdings könnte die Substratkohäsion am Untersuchungsort eine mechanische Obergrenze für die Thallusgröße darstellen. *F. distichus* Thalli widerstehen der Strömung nicht nur durch ihre Befestigung, sondern wir zeigen in Strömungskanalversuchen, dass sich die Thalli im Wasserfluss auch zum Substrat hin neigen und sich deutlich stromlinienförmig ausrichten, wodurch der Zug, dem die Befestigung ausgesetzt ist, verringert wird.

## Introduction

Seaweeds attached to the ocean shores are exposed to waves and currents. These impose much higher forces on them than would air of similar flow velocities due to the higher density of water (see exemplarily comparison by Denny and Gaylord 2002). While algae do benefit from the water moving around them, which transports dissolved gases and nutrients, removes waste products, and disperses gametes and spores (e.g., Koehl 1984; Koehl 1986), they also risk being torn or dislodged by the hydrodynamic forces (e.g., Burnett and Koehl 2022). Nevertheless, many seaweeds do not look as one would expect for organisms in a wave-swept environment, as has been pointed out by, for example, Denny and Gaylord (2002)—instead of being small, armored, and firmly attached to the ground such as limpets, for example, seaweeds are typically made from weak and compliant materials, they come in a variety of shapes that are not *per se* streamlined, and they sometimes reach giant sizes (stipes of the bull kelp *Nereocystis luetkeana*, for example, can reach 36 m in length, Druehl and Clarkston 2016). How then do they stay in place?

**Fig. 1:**
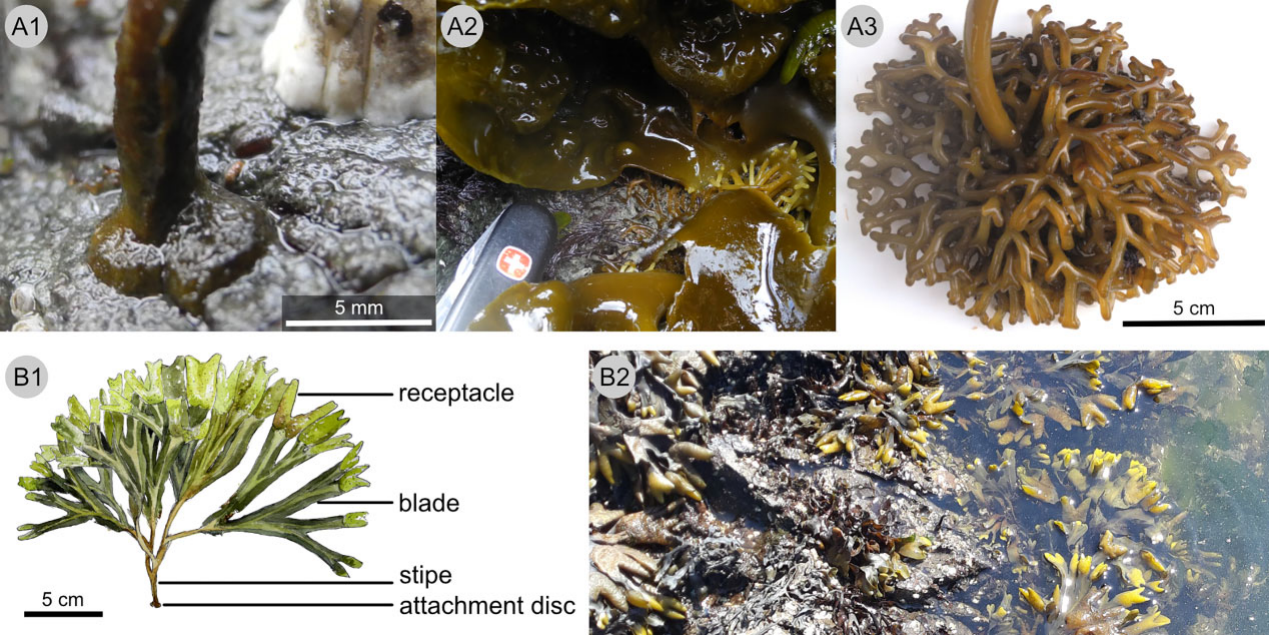
Holdfasts of marine macroalgae and habitus of *F. distichus*. Marine macroalgae attach to their substrates via for instance attachment discs (A1, *F. distichus*) or hapteral holdfasts (A2 *Hedophyllum sessile* and A3, *Nereocystis luetkeana*, drift specimen). The thallus of *F. distichus* is composed of the attachment disc, the stipe and blades that in a reproductive specimen bear fecund receptacles at their tips (B1). *Fucus distichus* growing in the rocky intertidal (B2).

On hard-bottom substrates, macroalgae are limited to a superficial attachment, as compared to deeply penetrating root-like filaments formed by mud-dwelling species such as *Rhipocephalus* sp. (Daweson 1966). Attachment is typically secured by hapteral holdfasts of different shapes (e.g., *N. luetkeana, Hedophyllum sessile*; Fig. 1B, C) or attachment discs (e.g., *Fucus distichus*  Fig. 1A). To resist the drag load imposed by the water flow, seaweeds can increase the break force of the attachment and the rest of the thallus (“tolerate drag”), but can also reduce the drag they experience (“avoid drag”) (cf. Starko et al. 2016). These traits are actually traded off in kelp species (Starko et al. 2016). Drag is reduced in several ways, thanks to the flexibility of the seaweed body, which is the key trait for macroalgae to persist in wave-swept environments (e.g., Koehl 1984; Koehl 1986; Denny and Gaylord 2002; Koehl 2022). Flexible macroalgae are passively reconfigured into a streamlined shape (Carrington 1990; Harder et al. 2004; Martone et al. 2012; de Bettignies et al. 2013); they may bend down toward the substrate where flow velocities are lower (Koehl 2000), and, if long enough, they can move with oscillating water flows until the flow reverses (Koehl 1984, Koehl 1986).

Ultimately, a macroalga will be dislodged if the hydrodynamic load surpasses the attachment’s break force. When the waves pull on the alga, it deforms in response, depending on its shape (cross-sectional area and length) and on the stiffness of its organs. The force that the water (or the experimenter) applies to the organs and the corresponding deformation give the so-called force–displacement curve. Normalized to stress (force per cross-sectional area) and strain (displacement relative to initial length), the slope of the curve represents the material property elastic modulus in tension. The area under the force–displacement curve until break represents the mechanical work required for breakage. This work can be large if an organ has a high absolute break force, but also if an organ is very extensible. In the latter case, a greater portion of the hydrodynamic energy is absorbed in stretching the organ. Thus, not only the alga’s absolute break force but also its stiffness and extensibility determine whether it breaks under a given hydrodynamic load or not (cf. e.g., Koehl 1984; Niklas 1992).

In macroalgae studies, mechanical properties are typically measured in the field in pull-to-break tests that record maximum performance only, or samples are transported to the laboratory where one can use materials-testing machines to measure force–displacement curves and thus also capture the loading response prior to break and derive a broader range of material characteristics (Burnett and Gaylord 2022).

In this study, we present a new method for continuous mechanical testing in the field and apply it to the attachment structure of *F. distichus*, that is, the disc and the lower stipe that connect the blades to the substrate. Our motivation for this biomechanical study of a marine attachment in the field is two-fold: One, it allows us to extract a detailed set of mechanical design features of interest from an engineering or materials science perspective as they may serve as biomimetic inspiration. Two, the presented setup may be used for studies that concentrate not only on biomechanical mechanisms but explore these in ecological contexts, “taking biomechanics to the field” (cf. Bauer et al. 2020).

*Fucus distichus* (formerly known as *F. gardneri*) (Fig. 1B) is an intertidal alga, which grows on rocks in wave-sheltered to moderately wave-exposed sites, and can be found on the North American west coast from Alaska to Santa Barbara, California (Druehl and Clarkston 2016). Thalli in the genus *Fucus* (Rockweed) are dichotomously and profusely branched, and the flattened branches form receptacles at their tips when the individual is reproductive (Druehl and Clarkston 2016; Hatchett et al. 2022) (see Fig. 1B). The receptacles contain conceptacles that release the gametes into the water where fertilization takes place; the zygotes then sink to the bottom, secrete an adhesive, and firmly attach to the substrate (for details see reviews by Tarakhovskaya 2014; Hatchett et al. 2022). The germling forms rhizoids equipped with adhesive mucilage, which grow across the surface and penetrate the substrate if possible (Hardy and Moss 1979; study on *F. spiralis*, *F. serratus*, and *F. vesiculosus*). As the germling develops further, hyphae grow down between the rhizoids, penetrate into the substrate or spread out over the rhizoids, and thereby form the attachment disc (Moss 1948; *F. vesiculosus*). Throughout the life of the alga, hyphae continue to grow and penetrate the outer borders of the disc, advance over the substrate, and increase the area of the attachment (Moss 1948; *F. vesiculosus*). When the thallus of *F. distichus* reaches 9.5–14.5 cm in length, it typically becomes reproductive for the first time (Ang 1991a) and may remain reproductive for prolonged periods during its life span of 2–3 years (Powell 1957; Ang 1991a). In the genus *Fucus*, reproduction typically occurs very close to the parent plant, as viable gametes and zygotes disperse only by a maximum of a few meters (reviewed in Hatchett et al. 2022). Even though dislodged thalli or parts thereof may continue to aid dispersal (reviewed in Hatchett et al. 2022), we expect a high selective pressure for an individual to remain attached, as dislodged individuals risk drifting to land and dying.

We measured force and displacement for the *in situ* dislodgement of *F. distichus* and analyze its failure behavior and failure modes (disc vs. stipe) to present a comprehensive mechanical characterization of its attachment. We compare our field results with results from laboratory tensile tests run on stipes and thereby isolate the mechanical behavior of one part of the attachment, to interpret the failure behavior of the entire structure. Furthermore, we take into account that mechanical traits are often not static but dynamic, and methodological details can affect the observed mechanical properties (Burnett and Gaylord 2022). In that respect, we expect that organs and tissues of *F. distichus* behave viscoelastically like many biomaterials (e.g., Niklas 1992). That is, they will largely return to their original shape after being loaded but this return is not instantaneous, and how strongly such a viscoelastic material deforms depends on time (Niklas 1992, 90). We thus expect that the mechanical properties of *F. distichus* stipes depend on the strain rate at which they are loaded, comparable to, for example, kelps (Burnett and Koehl 2021; *Egregia menziesii*). Strain rates used for dislodgement tests are recorded but vary due to manual handling, so we employed different fixed strain rates in the laboratory tensile tests for comparison. As we also expect that mechanical properties of *F. distichus* depend on age, comparable to the tissues of other algae species (Krumhansl et al. 2015; Burnett and Koehl 2019), we ran tensile tests on both young and mature stipes for comparison, and controlled for age in dislodgement tests, which we only carried out on young individuals. Due to methodological constraints, tensile tests yielded only the elastic modulus in tension, so unfortunately analysis of age- and strain rate dependency in this study is limited to this property, although other properties such as break force will be more decisive for macroalga dislodgement.

We link the mechanical analysis of the attachment to the thallus in its environment, elucidating if the risk of dislodgement due to drag dictates and limits thallus size at the sampled site. Our hypothesis is that one, the attachment’s break force scales with the expected load for the corresponding thallus. And two, that the break force scales with attachment size (stipe cross-sectional area, disc area, and disc wet weight). As a proxy for the expected load, we measured thallus size (planform area, biomass, and length), since we assume that drag scales directly with thallus size. Aside from size, drag also depends on the drag coefficient (e.g., Vogel 1994), that is, on thallus shape. However, we dislodged only young thalli of one species and from one growth site, resulting in low shape variation in the dataset. Moreover, we are interested in dislodgement, so drag force at extreme water velocities is relevant. It has been shown that at high water velocities, drag coefficients for different morphologies converge and drag force is mainly determined by size, namely thallus area and biomass (Carrington 1990; Haring and Carpenter 2007; de Bettignies et al. 2013). To find if there is a substrate specific constraint on thallus size, we compare thallus size distribution at the sampled site to the size distribution nearby.

While the attachment structure is crucial to withstand drag, we expect that *F. distichus* thallus properties will additionally mitigate the drag transmitted to the attachment. To explore how, we use flume experiments to measure thallus bending angle and projected thallus area at different water speeds.

Overall, in this study we elucidate (1) what are the main mechanical features of *F. distichus* attachment that prevent its dislodgement? And (2) does the risk of dislodgment due to drag dictate and limit thallus size at the sample site? Finally, we also illustrate (3) how *F. distichus* may mitigate drag load.

## Methods

### Field site

Laboratory tests were run at the Friday Harbor Laboratories on San Juan Island, Washington, USA. Field work was carried out in the rocky intertidal zone in front of the laboratories, where *F. distichus* is abundant. The area is protected from ocean swell but exposed to tidal currents, boat wakes and small wind waves, and a maximum water flow speed of 1.95 ± 0.24 m/s was measured during flow characterizations in August 2000 (Haring et al. 2002).

**Fig. 2:**
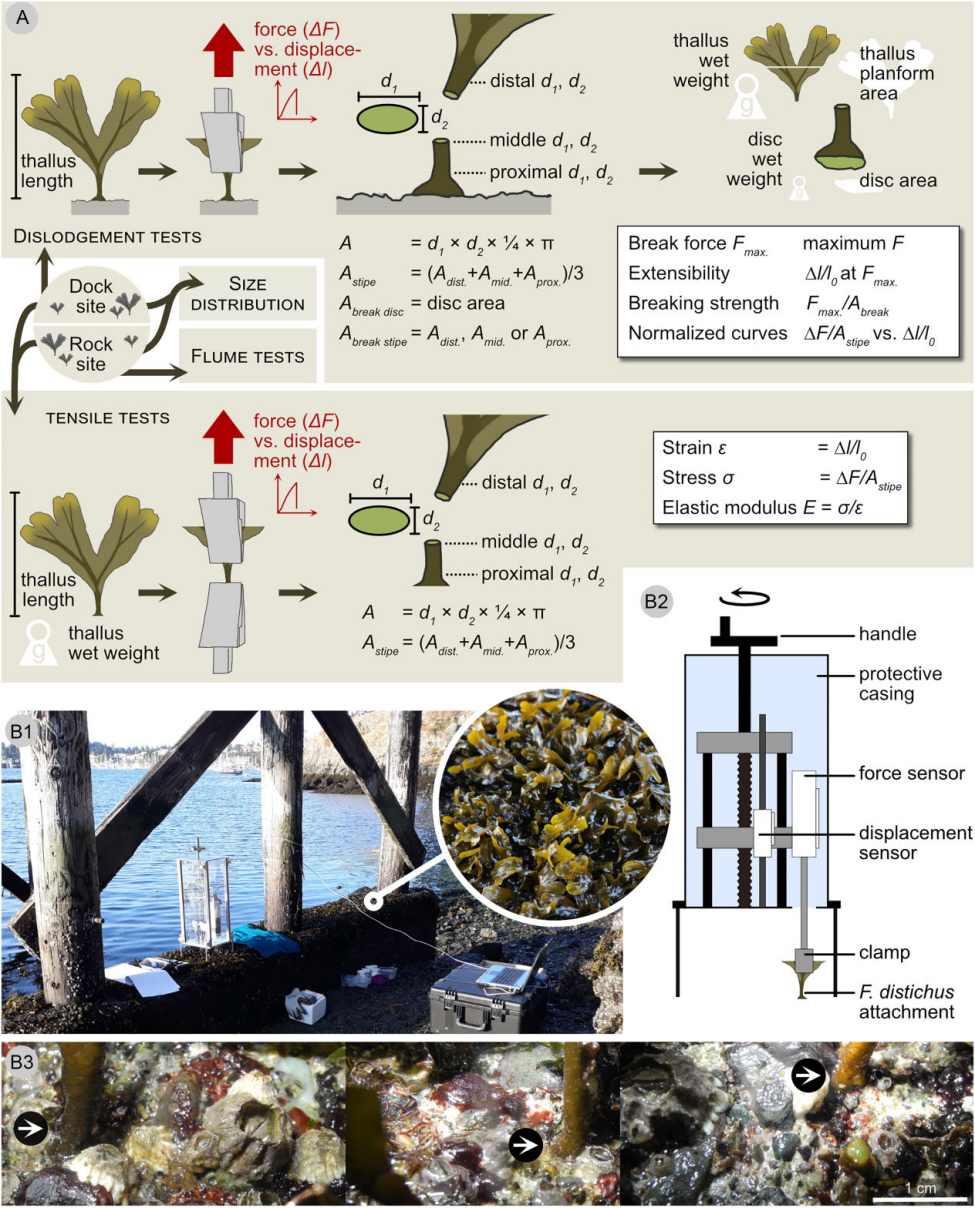
Study workflow and methodology. *Fucus distichus* thalli were subjected to dislodgement tests, tensile tests or flume tests. Depending on the experiment, tests were performed on thalli from two different sites (rock site and dock site), and thallus size distribution was assessed at both sites. For dislodgement and tensile tests, work flow and mechanical and morphological variables measured or derived from measurements are illustrated (see text for details) (A). *In situ* dislodgement tests were carried out at the dock site (B1), using a customized portable setup (B2). At this site, *F. distichus* attachment discs (arrows) anchored on a highly heterogeneous surface (B3).

Dislodgement tests were carried out on *F. distichus* growing on a concrete block supporting pillars of a bridge leading to the laboratories dock (“dock site”). The latter has a highly heterogeneous surface that incorporates different sized pebbles and is densely colonized not only by *F. distichus* but also by, for example, soft crustose algae, filamentous algae, and barnacles (Fig. 2B3). This site was used for establishment of the field dislodgement tests as it is accessible and has, on a macroscale, a flat surface (Fig. 2B1). Small specimens were abundant at the site, and these were more successfully clamped into the testing machine than larger specimens. To avoid bias within the dataset and control for a possible age effect, we tested only *F. distichus* without receptacles (“young thalli”), which reduced the size range of thalli (see Results section). Thallus size distribution was analyzed at the dock site and compared with the size distribution at surrounding rocks at the beach (“rock site”). Tensile tests were carried out on stipes of *F. distichus* collected from the dock site and the rock site, including both young specimen and specimen with receptacles (“mature thalli”). Analysis of a possible effect of collection site on organ or tissue mechanics lies beyond the scope of this study, but for methodological transparency, we report collection site with the data in the corresponding figure. In general, overall healthy thalli were chosen, with no more than minor traces of abrasion or wounding.

### Size distribution

To estimate the size distribution in the population, two plots of ca. 30 × 40 cm each at the rock site and the dock site were cleared completely (October 5th and 18th, 2018). Each thallus was measured in length using measuring tape and the number of thalli per size category small (<10 cm), medium (10–20 cm) and large (>20 cm) were counted. Suitable thalli were used further for tensile testing.

### Dislodgement tests

For field dislodgement tests, a customized portable material testing system was used (based on a system by Weinmann Sondermaschinen und Steuerungsbau GmbH, Hersbruck, Germany) (Fig. 2B2). The system is contained in a protective case and is equipped with a 500 N load cell (Sauter GmbH, Bahlingen, Germany). It tracks force, displacement, and time during the test with a time interval of 0.3 s. The setup is placed over the sample by adjusting the protective case’s four feet, and the sample is gripped in a metal clamp connected by a fixed rod to the load cell within the case. The load cell is moved upwards by manually turning a handle and thus pulls the sample away from the substrate perpendicularly.

A total of 38 dislodgement tests were carried out in August 2018, on a falling tide from early morning through midday. The canopy kept the samples hydrated, and in addition the testing area was covered with a towel damp with seawater to ensure that the attachment was hydrated when tested. Young thalli were chosen at the dock site and their length recorded to the closest 0.5 cm using measuring tape. For fixation in the material testing system, the thallus was shortened directly prior to positioning the system above the attachment. The stipe was clamped 2 cm above the substrate. Thalli were chosen that had their first dichotomy a minimum of ≈2 cm distal to the substrate, so the unbranched stipe was gripped below or at the dichotomy. Occasionally, specimen were gripped slightly above a dichotomy, or the disc gave rise to an additional secondary stipe, which was then ignored for testing. A small block of dry ice was briefly pressed to the metal clamp to enhance fixation of the stipe within the clamp. The stipe was pulled upward as evenly as possible until failure of the attachment. The extension wheel is hand cranked, so the test speed varied, though the aim was for a consistent cranking motion. The instantaneous test speeds were calculated by dividing distance covered between two position measurements by time as calculated from sampling frequency. Instantaneous test speed was divided by the initial length (2 cm) to calculate strain rates. The maximum strain rate was 0.24 s^−1^, and on average the strain rate was 0.04 ± 0.03 s^−1^ (average calculated without including strain rates of 0 s^−1^). If failure occurred at the stipe, the disc was removed manually for morphological characterization if possible.

After the test, the stipe diameter was measured using calipers, at distal, middle, and proximal position, in two orientations (*d_1_*, *d_2_*) (Fig. 2A). At each position, the cross-sectional area was approximated as an ellipse (*A* = ¼ × *d_1_* × *d_2_* × π), and for morphological characterization of the specimen, the average cross-sectional area was calculated. Thalli were stored in seawater and processed on the same day in the laboratory. They were flattened under a plastic sheet and photographed to measure planform area (Nikon Coolpix P7000, Nikon Corporation, Tokyo, Japan). Thalli were slightly blotted dry and weighted. The disc was cut off, weighted and photographed using a stereomicroscope, to measure its projected area (Zeiss SteREO Discovery.V20 microscope equipped with AxioCamHRc Zeiss Camera, and Software Axiovision, AxioVS40 4.8.2.0; Carl Zeiss AG, Oberkochen, Germany). Image analysis was performed in ImageJ (1.49i and 1.52a, National Institutes of Health). From the mechanical measurements, break force (maximum force *F*_max_*_._*) was retrieved. Work to break was calculated as the area under the force–displacement curves up to *F*_max_*_._*, by averaging the left and right Riemann sum. Breaking strength was calculated by dividing *F*_max_*_._* by breaking area. For thalli that broke at the disc, breaking area was estimated as the disc area measured on the stereomicroscope photos of the disc underside. For thalli that broke at the stipe, we took the cross-sectional area calculated based on the diameter measurements at either the distal, middle, or proximal position, depending on break location. If necessary, another set of diameter measurements was taken. Of note, breaking strength was thus calculated based on projected areas; however, the true breaking area will be slightly larger due to the rough surface of the breaks. Extensibility of the attachment structure was calculated by dividing displacement at *F*_max_*_._* by initial length (= 2 cm). Due to the very rough surface and the fact that the thalli do not stand erect but sometimes were a little slack in the clamp, the initial sample length could not be set with high precision. In consequence, the value for extensibility has a broad margin of error. However, even if initial sample length were to diverge by plus or minus 5 mm, we can still derive the order of magnitude of extensibility (for comparison: mean extensibility we measured ranges between 15 and 25% for assumed initial sample lengths between 1.5 and 2.5 cm). To be able to compare the force–displacement curves recorded during dislodgement with the stipe elastic modulus in tension, the curves were normalized as follows: The displacement was related to the initial sample length (2 cm) and the force was related to the stipe cross-sectional area.

### Tensile tests

A total of 71 thalli were collected from rock and dock site in July and October 2018. Thalli were chosen that had a minimum of 1 cm unbranched stipe, that could be used for testing. Thalli were cut in the field close to the disc and stored in a bucket of seawater or in running seawater prior to testing 2–6 h after collection. Overall length of the thallus was measured using measuring tape, and thalli were blotted to remove surface moisture and weighted. Thallus length was used as proxy for thallus age to analyze if elastic modulus changes with thallus age, because length as a continuous value provides more information than the categorical “young” or “mature.” Since *F. distichus* grows apically (e.g., Moss 1967), the basal stipe part usually will be older if the entire thallus grew longer. Tensile tests were performed using a material testing system equipped with a 500 N load cell (MTS Synergie 100, MTS Systems Corporation, Cary, NC, USA; run with Software TestWorks^®^ 4 Version 4.08 B). Stipes were cut from the thallus and clamped into the material testing system with a constant distance between clamps of 1 cm. The upper clamp usually gripped at or close to the first dichotomy. A small block of dry ice was briefly pressed to the metal clamp to enhance fixation of the stipe in the clamp. Stipes were pulled with a velocity of 100, 50, and 10 mm/min (the latter only for samples collected in October), which corresponds to strain rates of 0.17, 0.083, and 0.017 s^−1^. After the test, the stipe diameter was measured using calipers at distal, middle, and proximal position, in two orientations (*d_1_*, *d_2_*) (Fig. 2A). At each position, the cross-sectional area was approximated as an ellipse (*A* = ¼ × *d_1_* × *d_2_* × π), and the average cross-sectional area was calculated.

Fixation of the stipes in the clamps was challenging. Tests usually ended by break at the clamp or slip of the sample from the clamp. Break force, stress and strain could thus not be derived reliably. Force-displacement curves sometimes started with a short flat region and/or had small jumps in the beginning, which we attribute to the sample being a little slack or jerking a bit before proper grip was established. Prior to test end the curve often flattened, and we cannot exclude that slippage from the clamps is involved. To derive the elastic modulus, we therefore chose a linear region prior to the potential onset of slipping and after initial artefacts in the curve (see Supplementary Fig. S1). Force–displacement curves were transferred into stress (σ)–strain (ε) curves by relating the force *F* to the cross-sectional area *A* (*σ = F/A*) and the displacement *l* to the initial length *l_0_* (*ε = l/l_0_; l_0_* = 1 cm), with the displacement *l* referring to the extended length directly, not including the initial length. Of note, engineering stresses and strains and not true stresses and strains were calculated, neglecting the deformations during the test. The elastic modulus *E* was derived as the slope of the linear regression within the chosen region of the curve (*E = σ/ε*).

### Flume tests

Ten mature thalli were cut just above the disc from the rock site, stored in seawater and tested on the same or the following day. Thalli were clamped at their stipes to a metal frame, which was then lowered into a flume (flow visualization water tunnel, model 1520-H, Rolling Hills Research Corporation, El Segundo, Canada), so that the thallus floated upright in the water with the flat side of the stipe orientated perpendicular to the flow. The water speed in the flume was set to 0, 1, 3, 6, 10, 15, and 20 in/s (0, 0.03, 0.08, 0.15, 0.25, 0.38, and 0.51 m/s). One minute after each new setting, a picture of the specimen from downstream and one from the side was taken (Nikon Coolpix P7000, Nikon Corporation, Tokyo, Japan and Canon PowerShot D10 Digital Camera, Canon Inc., Tokyo, Japan). On the downstream pictures, the projected area was measured using ImageJ (1.49i and 1.52a, National Institutes of Health). As a measure for streamlining at different water flow speeds, the projected area in % of the projected area at 0 m/s is reported. Additionally, the projected area is reported in relation to the planform area. To measure planform area, thalli were flattened under a plastic sheet and photographed (Nikon Coolpix P7000, Nikon Corporation, Tokyo, Japan). On the side view pictures, the angle of the thallus relative to the flume bottom was measured, based on a visual selection of the main line along which branches and blades were orientated.

### Statistics

Either means ± standard deviations or medians with interquartile ranges (IQRs) are reported as measures for central tendency and dispersion of a variable. For comparisons between two groups, normality within groups and homoscedasticity of variances was checked via graphical assessment of a quantile–quantile plot and Levene’s test, using leveneTest from package car. If assumptions were not met, a Wilcoxon rank-sum test was performed instead, using wilcox.test from package stats.

For comparisons between three groups, an analysis of variance (ANOVA) was performed, using aov from package stats. Assumptions of normality of residuals and homoscedasticity of variances were controlled via graphical assessment of a quantile–quantile plot of residuals and a residuals-vs.-fitted plot. If assumptions were violated, a Kruskal–Wallis test was employed, using kruskal.test from package stats. Test sensitivity was assessed by estimating the effect size the ANOVA would detect with a test power of 0.8 (significance level alpha = 0.05), using pwr.anova.test from package pwr. If group sizes were extremely unbalanced, two sensitivity calculations were made using the maximum and minimum group size to estimate a range of effect sizes that could be detected. As a general orientation, effect sizes were interpreted as small, medium, and large for *f* = 0.10, *f* = 0.25, and *f* = 0.40, following Cohen (1988, 284 ff).

Linear correlation between two variables was measured by calculating Pearson’s correlation coefficient *r* and corresponding *P*-value, using the function cor.test from package stats. As a general orientation, correlation coefficients of approximately *r* = 0.10, *r* = 0.30, and *r* = 0.50 were interpreted as small, medium, and large effects, respectively, following Cohen (1988, 79 ff). Test sensitivity was assessed by calculating the correlation coefficient *r* that would be detected with a test power of 0.8 and significance level alpha = 0.05, using pwr.r.test from package pwr. The dataset from dislodgement tests contained one very large thallus compared to the rest of the samples (length 17.5 cm). To check if correlations were robust and not depending on the influence of only that one extreme data point, correlations were re-calculated omitting this data point for comparison.

To find whether there is a preferred failure location within the attachment structure (disc or stipe, see results), a two-sided binomial test was employed (biom.test from the package stats), using *P* = 0.5 as null hypothesis (i.e., a similar probability for the two failure locations). Test sensitivity was assessed by calculating the effect size and thus ratio of failure locations that the test would detect as diverging from a 50:50 ratio of failure locations with a test power of 0.8 and significance level alpha = 0.05, using pwr.p.test and ES.h from package pwr.

The following significance levels were applied: *P*-value ≥ 0.05, not significant (n.s.); *P*-value < 0.05 ≥ 0.01, significant (∗); *P*-value < 0.01 ≥ 0.001, high significant (∗∗); and *P*-value < 0.001, highly significant (∗∗∗). All analyses were conducted using R (version 3.6.2) (R Core Team 2019).

## Results

### Thallus size distribution at sampling site

**Fig. 3:**
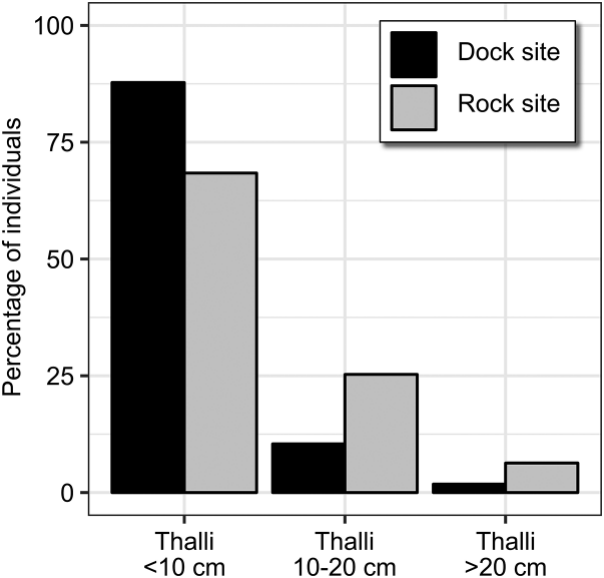
Thallus size distribution. Percentage of small (<10 cm in length), medium (10–20 cm), and large (>20 cm) thalli in two plots each at the dock site and the rock site. Total number of thalli 499 (dock site) and 174 (rock site).

At both locations, small thalli (<10 cm) were more common than medium sized (10–20 cm) and large (>20 cm) thalli (Fig. 3). At the dock site, 88% of thalli were small, 10% medium sized, and 2% large. Compared to that, at the rock site there were fewer small thalli (68%), but more medium sized (25%) and more large ones (6%).

### Dislodgement tests

**Fig. 4:**
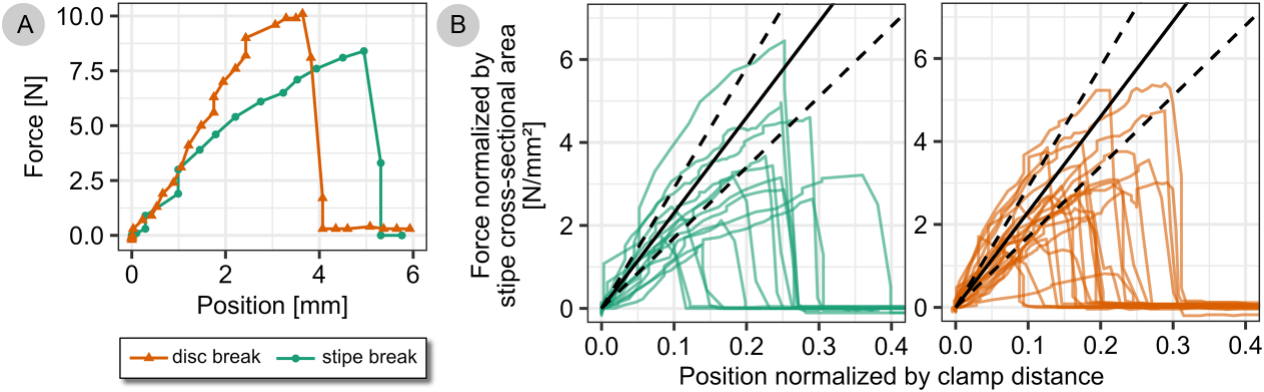
Force–displacement curves of *F. distichus* dislodged in the field. Example curves for a thallus that broke at the disc (triangles) and one that broke at the stipe (circles) are shown (A). Force–displacement curves were normalized by relating force to stipe cross-sectional area and position to initial distance between substrate and clamp. Normalized curves are shown separately for all thalli that broke at the stipe (left plot) and the disc (right plot), together with a reference line with a slope corresponding to the elastic modulus measured for young stipes in tensile tests (23 ± 6 N/mm², dashed line shows standard deviation) (B). All curves are shown separately in Supplementary Fig. S3.

**Fig. 5:**
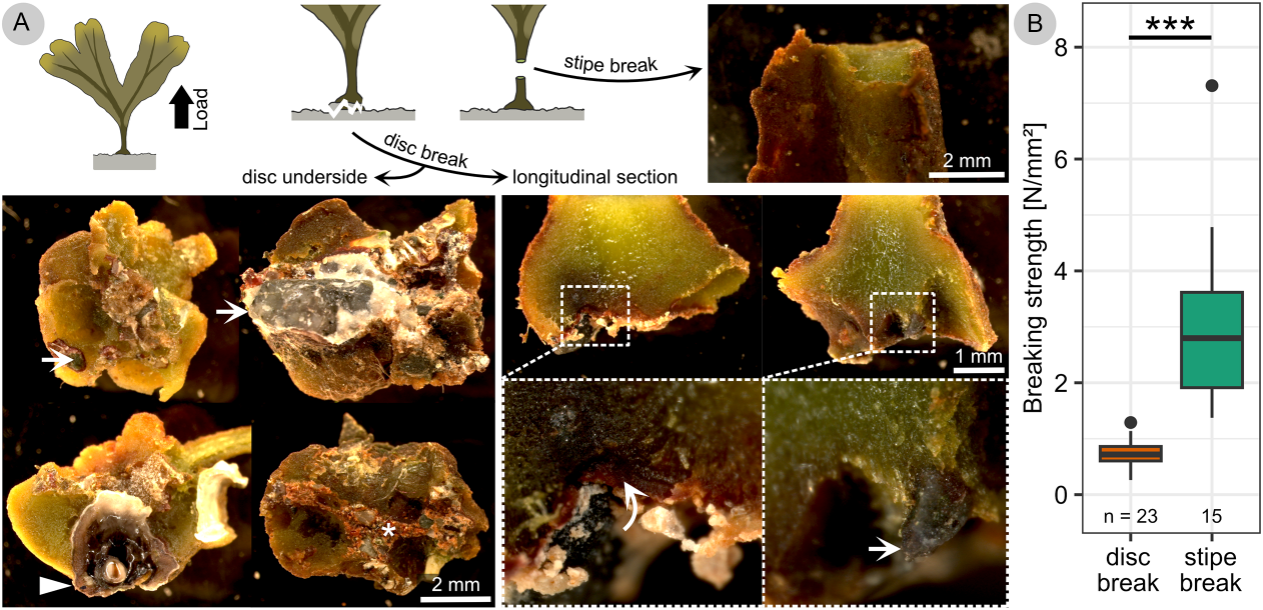
Failure habitus and breaking strength of *F. distichus* dislodged in the field. Stereomicroscopic photographs show breaking areas of the attachment of *F. distichus* at stipe or disc. Dislodged discs carried with them considerable amounts of substrate, including gravel (arrowheads), barnacles (triangle), sand grains (asterisk), or parts of other algae (curved arrow) (A). Breaking strength was significantly higher for samples that broke at the stipe than for samples that broke at the disc (Wilcoxon test, *P* = 1 × 10^–10^) (B).

**Fig. 6:**
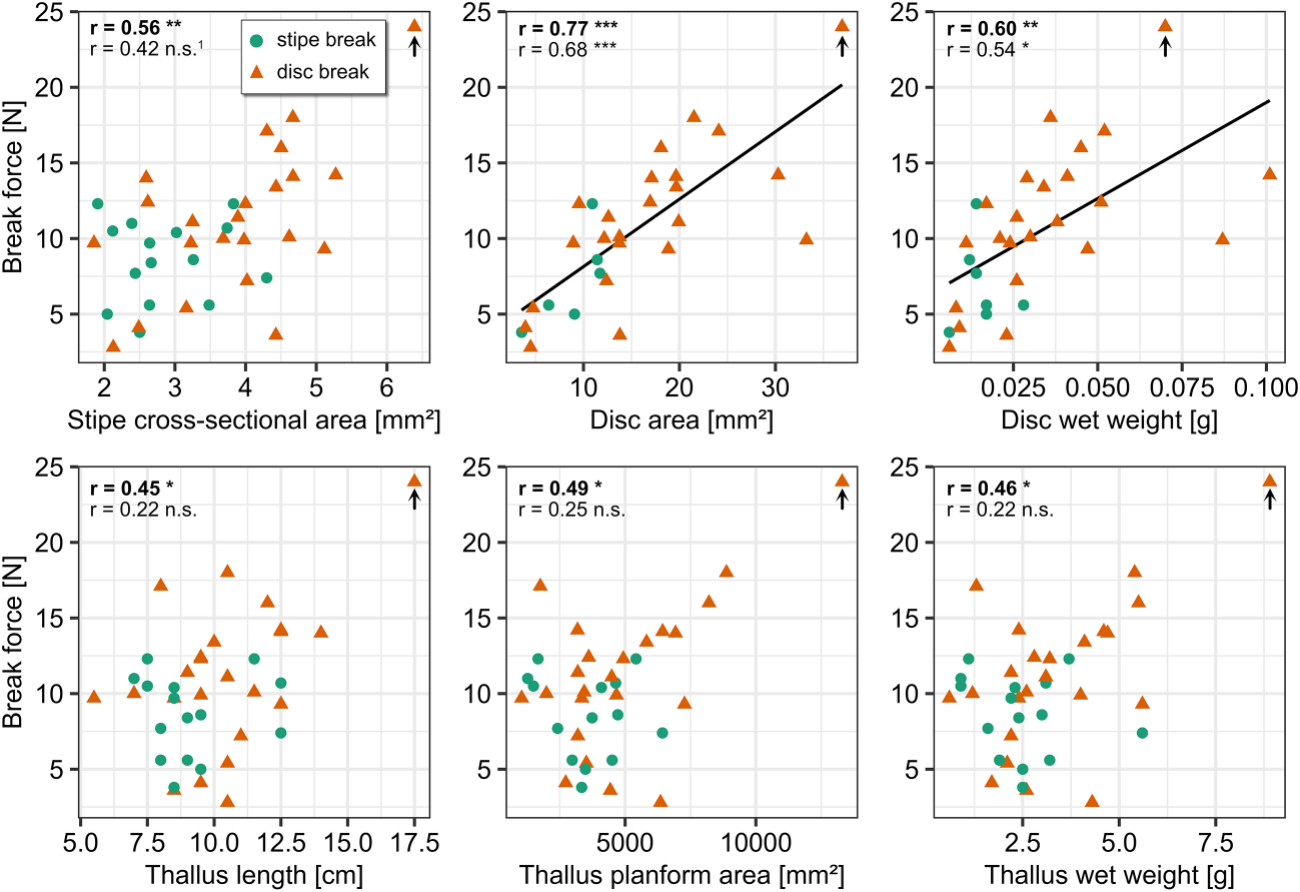
Break force and morphology of *F. distichus* dislodged in the field. Break force is plotted against attachment size (stipe cross-sectional area, disc area, and disc wet weight) and thallus size (length, planform area, and wet weight) for both failure locations, stipes (circles) and discs (triangles). Correlation coefficient Pearson’s *r* is indicated in the plot in bold, and in normal font re-calculated without one extreme value, marked by an arrow. Significance levels of Bonferroni-corrected *P*-values are indicated (^1^correlation coefficient significant prior to Bonferroni-correction). Correlations that remain significant even if the extreme value is removed are indicated by a regression line. *P*-values are indicated as follows: *P*-value ≥0.05, not significant (n.s.); *P*-value < 0.05 ≥ 0.01, significant (∗); *P*-value < 0.01 ≥ 0.001, high significant (∗∗); and *P*-value < 0.001, highly significant (∗∗∗). See text and Supplementary Table S1 in supplement for detailed statistics results.

During dislodgement of the attachment of young *F. distichus*, the force generally increased in good approximation linearly up to a sudden drop (see Fig. 4 and Supplementary Fig. S3). Sometimes the curve flattened shortly before failure. When normalized by the stipe cross-sectional area and the initial sample length, the slope of the curves is in the order of magnitude of the elastic modulus measured for the stipes (Fig. 4B). During the tests, the stipes elongated until they suddenly broke or until the disc came off the substrate abruptly. Ripped off discs typically carried with them considerable amounts of substrate such as sand grains, gravel, barnacles, or parts thereof, or red algae material (Fig. 5A). The spot from which the thallus was dislodged was often not visible within the extremely heterogeneous and wet substrate, but sometimes remains of the disc tissue were detected on the substrate. Break force was 10 ± 4 N, extensibility 19 ± 8% and failure energy 25 ± 16 mJ. Thalli broke more commonly at the disc (*n* = 23, 61%) than at the stipe (*n* = 15, 39%), but this difference was not significant (binomial test, *P* > 0.05). Test sensitivity was such that a true ratio of 72:28 would have been detected with test power 0.8. Breaking strength was significantly higher when the thallus broke at its stipe (3.0 ± 1.5 N/mm²) than when it broke at its disc (0.7 ± 0.3 N/mm²) (Wilcoxon test, *P* = 1 × 10^–10^) (Fig. 5B). Thallus break force did correlate strongly with attachment size (stipe cross-sectional area disc wet weight, disc area; (*r* = 0.56, 0.60, 0.77, respective) (Fig. 6). For disc size, these correlations were robust even if re-calculated without one extreme value, for stipe cross-sectional area the re-calculated correlation coefficient was not significant after Bonferroni-correction. Break force also correlated strongly with thallus size (length, area, and wet weight), but these correlations only depended on one extreme value and did not remain significant if re-calculated without this value. Test sensitivity was such that large effect sizes could be detected reliably (*r* between 0.44 and 0.49 for alpha = 0.05, and *r* between 0.53 and 0.59 for Bonferroni-corrected alpha = 0.05/6). See Supplementary Table S1 in supplement for detailed statistics results.

Thalli were on average attached via a stipe of 3.5 ±1.1 mm² cross-sectional area and a disc of 15.2 ± 8.4 mm² area that weighted 0.03 ± 0.02 g. These morphological variables correlated strongly and positively with one another (*r* between 0.73 and 0.92; see Table 1 and Supplementary Fig. S4). Thalli were on average 10 ± 2 cm long, weighted 3 ± 2 g and had a planform area of 4404 ± 2431 mm². These morphological variables correlated very strongly with one another (*r* between 0.82 and 0.98). Stipe cross-sectional area correlated with thallus size (*r* between 0.62 and 0.65). These correlations were robust and remained strong and significant even if re-calculated without one extreme value and after Bonferroni-correction for multiple testing (see Table 1 and Supplementary Table S2 for detailed statistics results). Disc area correlated strongly with thallus size (*r* between 0.50 and 0.54). However, for thallus area, the correlation did not remain significant after Bonferroni-correction. For thallus length and wet weight, it did remain significant after Bonferroni-correction, but the correlations only depended on one extreme value and did not remain significant if re-calculated without this value. Test sensitivity for alpha = 0.05 was such that large effect sizes (*r* between 0.44 and 0.49) could be detected reliably, but for the Bonferroni-corrected alpha = 0.05/15 only very strong correlations could be detected (*r* between 0.56 and 0.63).

**Table 1:** Thallus and attachment sizing of *F. distichus* dislodged in the field. Correlation coefficient Pearson's *r* is reported for correlations between morphological characteristics of thallus and attachment in bold, and in normal font calculated without one extreme value (see Methods section for details). Significance levels of Bonferroni-corrected *P*-values are indicated. Sample size *n* = 38 with the following exceptions: thallus planform area *n* = 37, disc wet weight *n* = 30, and disc area *n* = 29. *P*-values are indicated as follows: *P*-value ≥0.05, not significant (n.s.); *P*-value < 0.05 ≥ 0.01, significant (∗); *P*-value < 0.01 ≥ 0.001, high significant (∗∗); and *P*-value < 0.001, highly significant (∗∗∗). See Supplementary Fig. S4 and Table S2 for correlation plots and detailed statistics results.

	Thallus wet weight	Thallus area	Stipe cross-sect. area	Disc area	Disc wet weight
Thallus length	0.85***	0.82***	0.65***	0.54*	**0.49 n.s.^1^**
	0.77***	0.73***	0.55**	0.35 n.s.	0.40 n.s.^1^
Thallus wet weight		0.98***	0.64***	0.55*	**0.41 n.s.^1^**
		0.97***	0.52*	0.35 n.s.	0.29 n.s.
Thallus area			0.62***	**0.50 n.s.^1^**	**0.34 n.s.**
			0.49*	0.26 n.s.	0.18 n.s.
Stipe cross-sect. area				0.73***	0.67***
				0.65**	0.62**
Disc area					0.92***
					0.93***

1 Correlation coefficient significant prior to Bonferroni-correction for multiple testing.

### Tensile tests

**Fig. 7:**
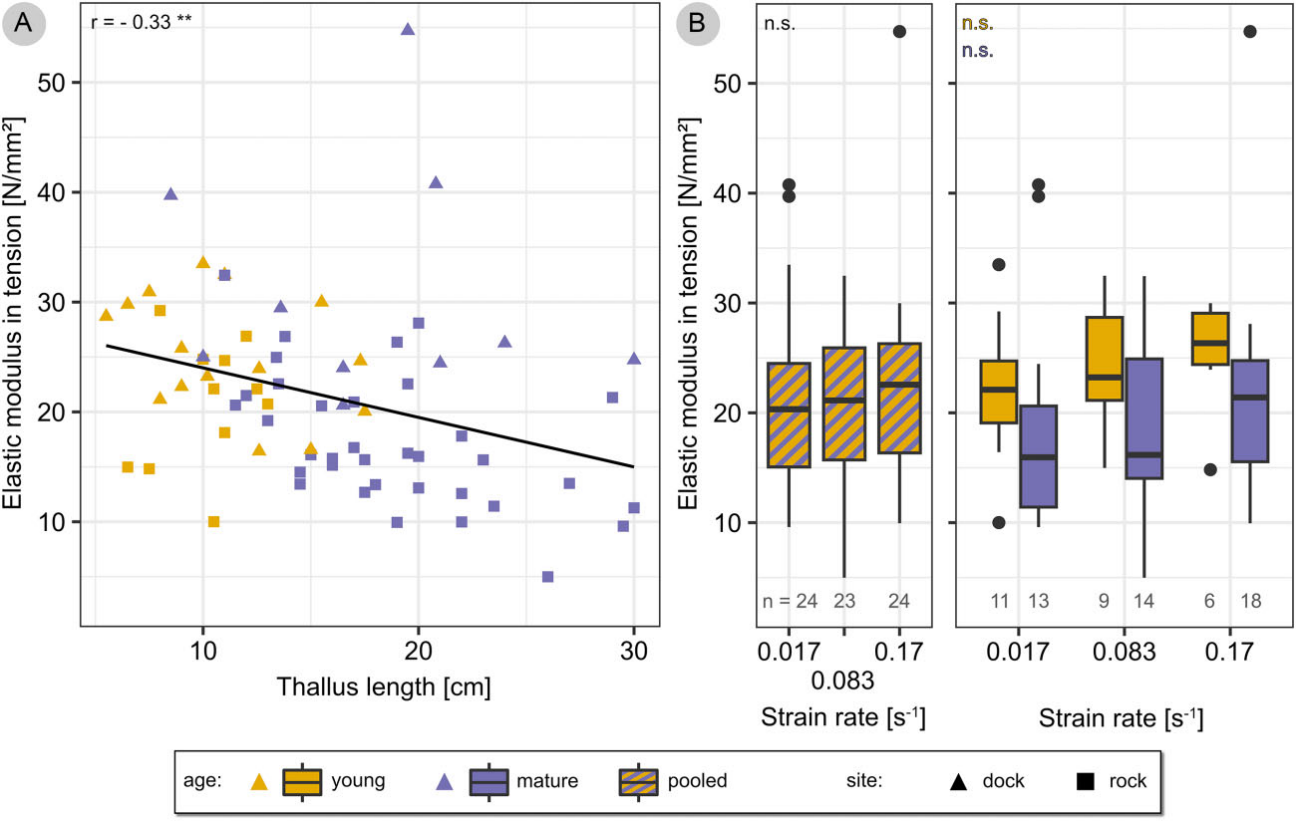
Elastic modulus in tension. Tensile tests were carried out on stipes of young and mature *F. distichus* thalli, using different strain rates. The elastic modulus is plotted against thallus length for young (yellow) and mature (purple) thalli. (A) A significant negative correlation was found (Pearson’s *r* indicated in plot). For methodological completeness, collection site is specified (samples collected from dock site as triangles, samples from rock site as squares). The elastic modulus is plotted for each strain rate pooled for young and mature thalli (hatched boxplots), and separately for young and mature thalli (yellow and purple boxplots, respectively) (B). Statistical analysis of the pooled data did not show a significant effect of strain rate on elastic modulus, and neither did it within each age group (see text for details).

The elastic modulus in tension was measured on stipes of young and mature *F. distichus*, using three different strain rates. Mature thalli were longer (18 IQR 7.5 cm) and heavier (20.1 IQR 26.9 g) and had thicker stipes (7.9 IQR 4.7 mm²) than young thalli (10.5 IQR 4.3 cm, 2.2 IQR 4.3 g, and 3.7 IQR 2.3 mm²) (Supplementary Fig. S2; Wilcoxon test, *P* = 2 × 10^–8^, *P* = 3 × 10^–9^, and *P* = 1 × 10^–8^, respectively). There was a moderate negative correlation between the stipe’s elastic modulus and thallus length (Pearson’s *r* = –0.33, *P* = 0.005) (Fig. 7A).

No effect of strain rate on elastic modulus pooled for young and mature thalli was found (Fig. 7B), according to an ANOVA (*P* > 0.05) and neither according to a Kruskal–Wallis test (*P* > 0.05), that was calculated since the assumption of normally distributed residuals was violated. Test sensitivity was such that a large effect size would have been detected reliably (effect size *f* = 0.38 for three groups with *n* = 23 and power = 0.8). A visual inspection of the elastic modulus evaluated separately by age shows a trend of increasing elastic modulus with increasing strain rate for both young and mature thalli (Fig. 7B). This effect of strain rate on elastic modulus is statistically not significant (ANOVA and Kruskal–Wallis test *P* > 0.05). Due to the very small sample sizes in sub-groups, test sensitivity was very low, however, so that only extremely large effect sizes would have been detected reliably (for young stipes with a varying group size of *n* = 6–11 the detectable effect size with power 0.8 ranges between *f* = 0.81–0.57; and for mature ones with a varying group size of *n* = 13–18 between *f* = 0.52–0.44).

The elastic modulus pooled for young and mature thalli and for all strain rates was 21 ± 8 N/mm² (21 MPa). The elastic modulus for stipes of young thalli was 23 ±6 N/mm² (pooled for all strain rates); this value was used for comparison with the results of dislodgement tests run on young thalli (Fig. 4).

### Flume tests

**Fig. 8:**
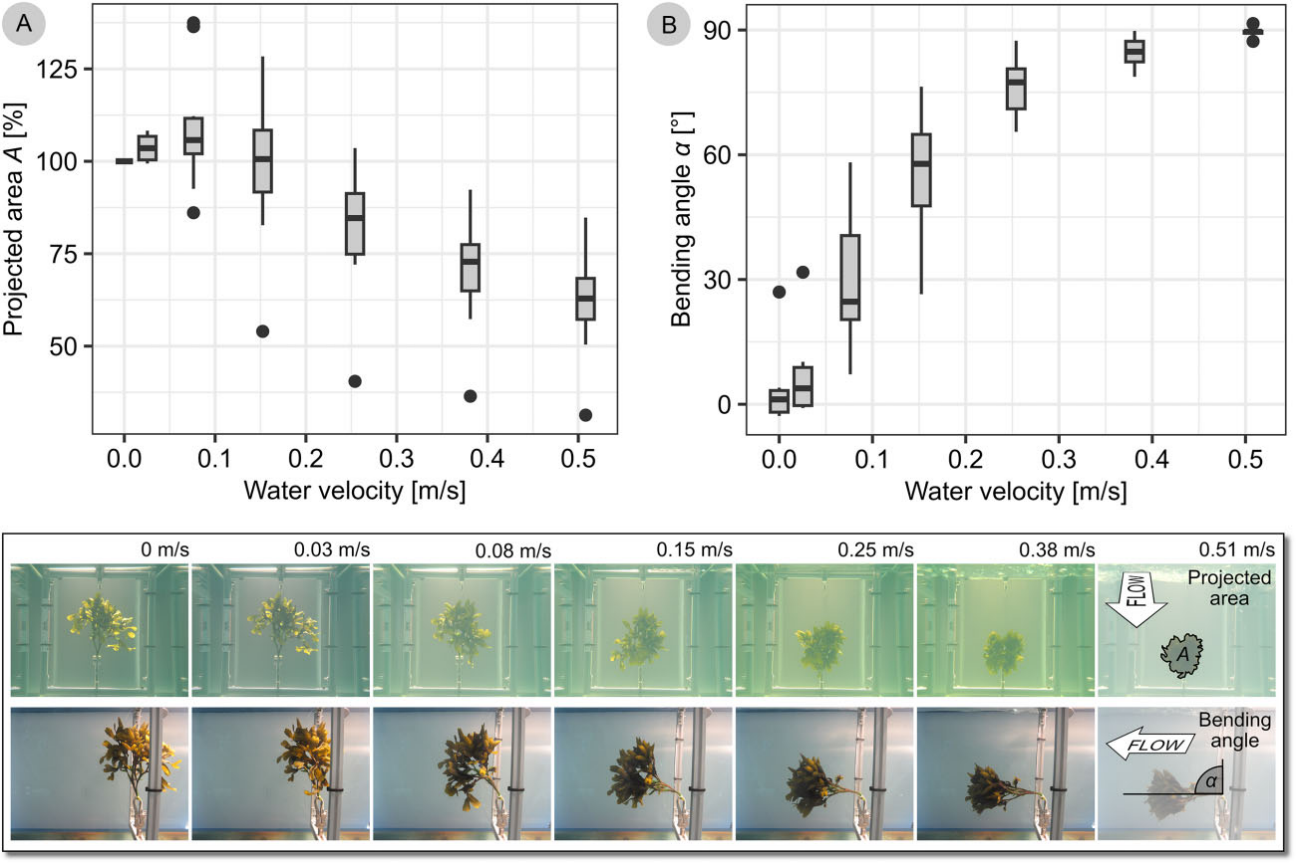
Streamlining and reorientation of *F. distichus* in water flow. A total of 10 thalli were subjected to increasing water flow speeds. Their projected area as viewed from upstream in % of the projected area at 0 m/s is shown in (A) and their bending angle in (B). Measurements on one exemplary thallus are shown below the plots.

The projected area of thalli subjected to slow water flow (0.03, 0.08 m/s) typically increased. In faster flow, the thalli streamlined considerably, so that at a water speed of 0.51 m/s, their projected area measured only 63% with an IQR of 11% of their projected area at 0 m/s (Fig. 8A) (*n* = 10). In relation to their planform area, this was 29% with IQR 5%. Moreover, thalli bent down parallel to the flow, and the bending angle at 0.51 m/s was 90.0 IQR 0.5° (Fig. 8B).

## Discussion

We successfully introduced a new setup for biomechanical field studies on macroalgae. Due to the manual handling, experiments were carried out at variable strain rates. This is similar to the thallus in its natural surroundings, where it is exposed to a variable water flow. Strain rates experienced by a seaweed can be estimated based on *in situ* force measurements (e.g., Gaylord 2000; Stevens et al. 2002; Gaylord et al. 2008; cf. also Burnett and Koehl 2021). Unfortunately, such measurements have not been carried out for *Fucus* species so far, and it therefore remains open how the strain rates used in this study relate to the actual strain rates experienced by *F. distichus* in the field. Tensile tests show a trend of increasing elastic modulus for increasing strain rate, but this was not significant. However, test sensitivity was very low, so we cannot preclude that there is an effect. We deem it reasonable to discuss our results from dislodgement tests in general without addressing viscoelastic effects, despite the varying strain rates used during the tests. For more in-depth comparative studies, we suggest to add a simple motor to the setup to enable strain-controlled experiments. Moreover, we did find that the elastic modulus in tension decreases with thallus age and our study thus highlights the importance to take organ age into account when designing and interpreting biomechanical studies (cf. Burnett and Gaylord 2022). The new setup we used in this study is more time consuming to handle than the standard spring scale (cf. Burnett and Gaylord 2022), and has limited precision compared to typical laboratory materials-testing machines, especially concerning the determination of maximum extensibility. However, it allows us to continuously and *in situ* record force and displacement during macroalgae dislodgement, and in consequence, we here not only discuss the maximum performance of the attachment, but subsequently evaluate the failure behavior in detail and provide a more complete picture of the attachment’s mechanical features.

Break (or dislodgement) force of 10 ± 4 N measured in this study is in an order of magnitude common for the genus *Fucus*, as shown by the following comparisons. Studies carried out at different locations with varying exposure, substrate types and directions of loading found break forces of <5–25 N (*F. vesiculosus* and *F. spiralis*, extracted from Fig. 3A in Jonsson et al. 2006), 3.5–8.4 N (*F. vesiculosus*; Thomsen 2004), 34–76 N (*F. vesiculosus*; Malm et al. 2003), and 18–34 N (*F. evanescence* and *F. serratus*; Wikström et al. 2002). Moreover, the measured dislodgement force matches the force expected based on the planform area of the measured thalli according to a relation found in a meta analysis by Thomsen and Wernberg (2005), which included 118 seaweed populations across different orders and habitats: For our thalli of 4404 mm² planform area, the expected break force calculates as 10.04 N. Our study thus confirms that across populations, thallus size is a good predictor for a seaweed’s break force.

Break force is determined by the organ’s breaking strength and cross-sectional area. In our dislodgement tests, we measured breaking strengths of 3.0 ± 1.5 and 0.7 ± 0.3 N/mm² for specimen that broke at the stipe or the disc, respectively. This matches results of previous studies, which report for instance a breaking strength of 4 N/mm² (extracted from Fig. 6 in Haring et al. (2002), only stipe breaks) and 0.5–1.5 N/mm² (extracted from Fig. 3 in van Tamelen and Stekoll (1997), mostly holdfast breaks). Our results show that for a brown algae, *F. distichus* has rather strong (but thin) stipes, compared to for example *Postelsia palmaeformis* (breaking strength of 1 N/mm^2^; Holbrook et al. 1991) or *Durvillaea antarctica* (breaking strength of 0.7 N/mm^2^; Koehl 1979). Compared to many red macroalgae, *F. distichus* has weak (but thick) stipes. For *Chondrus crispus* a breaking strength of 11 N/mm² was measured and for *Mastocarpus stellatus* 19 N/mm² (Dudgeon and Johnson 1992) (cf. Martone 2007). All of these algae materials are weak compared to typical wood which has a breaking strength of 100 N/mm² if measured along the grain (Niklas 1992, 104), and also compared to many other biomaterials (see e.g., summary by Koehl 1986).

The attachment failed more commonly at the stipe than the disc, but this difference was not significant, which implicates that there is no extremely strong (or weak) link in the attachment. *Fucus* species are capable of regeneration from remarkably small remains (McCook and Chapman 1992; Malm et al. 1999; *F. vesiculosus*, *F. evanescens*), and the more biomass remains, the more is regenerated (Kiirikki and Ruuskanen 2009; *F. vesiculosus*). Thus, a remaining stump should be more advantageous than a small piece of disc. However, for *F. distichus*, regeneration so far has been reported only for very small individuals (Ang 1991b); discs or stumps of larger individuals eventually disappear. This indicates that for *F. distichus*, failure of the attachment is typically fatal, no matter the exact failure location.

At a microscopic scale, it is highly unlikely that dislodgement is caused by adhesive failure of the disc, since studies on *F. vesiculosus* show that discs not only attach superficially, but hyphae from the discs penetrate wood (Moss 1948), other seaweeds (Moss 1948), barnacle shells (Barnes and Topinka 1969), and even bedrock (Morrison et al. 2009). Our investigations of the dislodged discs show that they grew over or in every crevice or loose part of the highly heterogeneous substrate at the dock site, providing tight form closure. They typically rip off parts of the substrate with them when they detach. This suggests that rather than pure adhesive failure, combined cohesive failure of substrate and of disc tissue occurs. Discs have a particular tissue structure compared to the rest of the thallus (Moss 1948; *F. vesiculosus*), but it remains an open question if this decreases tissue strength. It is more likely that weak or loose parts within the substrate limit the attachment rather than the strength of the disc. Accordingly, breaking strength was less than half for thalli that failed at the disc than for thalli that failed at the stipe.

*Fucus distichus* cannot control its growth location. Some kelp species possess motile spores that are chemotactic, which probably increases chances to settle in a suitable location (at least on a millimeter scale) (for details see review by Amsler and Neushul 1989; Schiel and Foster 2006), but for *F. distichus* the location, and thus the substrate, is determined by where the zygote falls (cf. discussion by van Tamelen and Stekoll 1997). The broadening of the stipe into a disc increases the contact area with the substrate as compared to the cross-sectional area of the stipes (discs have on average a 4.3 times higher area than stipes) and probably compensates somewhat for a potentially weak substrate. Accordingly, we found that larger discs provide a higher attachment force. This assures that even on the highly heterogeneous substrate, encompassing weak or loose material, the failure modes are not extremely unbalanced.

Of note, failure behavior may also depend on the angle at which load is applied to the structure. For standardization purposes, we pulled perpendicular to the substrate, but our flume experiments show that even at moderate water flows, the thallus bends down, that is, load will be transduced to the disc at an angle <90°. With this more parallel loading, we expect a higher proportion of disc failures (cf. van Tamelen and Stekoll 1997; Haring et al. 2002).

In addition to break force, the work required to remove the alga depends on the alga’s extensibility, that is, the further it has to be stretched, the more work is required. This extension can occur in a material’s elastic range but also beyond. Material tests on rachis tissue of the brown seaweed *Egregia menziesii* for instance have shown that the tissue extends far beyond its yield point, that is, plastic deformations contribute considerably to energy absorption (Burnett and Koehl 2021, Fig. 2). For the attachment of *F. distichus*, energy is mainly absorbed by elastic deformation of the stipe, as can be deduced from the force–displacement curves that closely match the stipe-extension curves in slope and show no, or very short, flat regions prior to failure that can be attributed to plastic deformation. The elastic modulus was 21 ± 8 N/mm², and thus smaller than what Haring et al. (2002) measured for *F. distichus* (38.30 ± 9.12 N/mm²). Such low moduli are typical for algae materials that are very compliant compared to other biomaterials. A stiffness of 1–100 N/mm² is characteristic for wet algae materials (Denny and Gaylord 2002; Harder et al. 2006; Demes et al. 2020), whereas the elastic modulus of wood ranges at 5000–15,000 N/mm² (Niklas 1992, 114), and also stems of many herbaceous plants are markedly stiffer than the algae stipes (50–1900 N/mm^2^; Niklas 1995). Fully desiccated however, algae tissues exhibit material properties in a range closer to wood and stiff leaves (Demes et al. 2020). Fracture of the attachment was sudden with smooth fracture surfaces rather than ductile, that is, without considerable plastic deformation, and occurred at 19% extension. This is higher than 9% extension as measured by Haring et al. (2002) and may be a little overstated due to initial slack in the samples. Still, the order of magnitude is similarly high to other algae stipes, which fracture at extensions of 9% (*Lessonia nigrescens*; Koehl 1979), 17% (*D. antarctica*; Koehl 1979), and 18–21% (*P. palmaeformis* stipe tissue; Holbrook et al. 1991). In their work on the giant kelp *Nereocystis luetkeana*, Koehl and Wainwright (1977) found an extensibility of 38% and suggest that this extreme extensibility is allowed by the 60° crossed helical arrangement of cellulose fibrils in the cell walls. Maybe similar ultrastructural properties enable high extensibility in *F. distichus*, but this still needs to be investigated. If the stipes had the same break force but were less extensible, work to break would be lower, that is, the stipes would absorb less hydrodynamic energy before they break. We expect that extensibility makes them more resilient against very short duration peak loads (“shock absorber,” cf. Koehl 1984).

More importantly, we expect that the stipes are not only highly extensible and compliant in tension, but also highly flexible in bending and torsion, which will allow them to reduce the drag they experience. For long macroalgae such as *N. luetkeana*, high extensibility and flexibility allow the seaweed to “go with the flow,” that is, it can deform with the flow so that the load is not (fully) transmitted to the attachment before the flow peak is over (e.g., overviews by Koehl 1984, 1986; Denny and Gaylord 2002). For the much shorter and intertidal macroalgae *F. distichus*, this particular mechanism of drag avoidance is probably less important (cf. experiments Friedland and Denny 1995 on *E. menziesii*).

Our flume experiments illustrate that *Fucus* thalli do mitigate drag in other ways: In water flow, their projected area reduces considerably, already at water velocities far below the peak velocities measured in the area, which were 1.95 ± 0.24 m/s (Haring et al. 2002). At water velocities of 0.5 m/s, the projected area was only 29% of the planform area, which is consistent with measurements on other non-coralline algae (Martone et al. 2012, Fig. 2). Reducing the area will reduce the drag *F. distichus* experiences. Concomitantly, the thallus may also change in shape to reduce the drag coefficient. In some species of algae, one behavior appears to be more pronounced, while in others, the other behavior is more pronounced (Martone et al. 2012). Bladed algae, for example, have been shown to be generally “shape changers,” while a branched coralline algae was shown to be an “area reducer”; other types are intermediate (Martone et al. 2012). To discuss these behaviors for *F. distichus*, future flume experiments need to include force data. This would also allow to check the assumption that the experienced drag scales directly with thallus planform area. Our flume experiments illustrate that in addition to area reduction, thalli bend down close to the substrate, which may reduce drag further (Koehl 2000; study on *Chondracanthus exasperatus)*. As typical for seaweeds (e.g., Denny and Gaylord 2002), flexibility thus seems to be a key feature for *F. distichus* to avoid hydrodynamic forces.

Unlike our expectation, we did not find an unequivocal and robust correlation between break force and thallus size; that is, larger thalli did not consistently attach more strongly. This seems to be because larger thalli did have thicker stipes, but not unequivocally larger attachment discs (corresponding correlations were not robust if one extreme value was removed). Moreover, thicker stipes did not as clearly provide a higher break force as expected. Perhaps other effects determining the break force dominated the relation between stipe cross-sectional area and break force, for example small natural variations in organ strength between individuals, or a varying degree of organ damage which affects strength both instantaneously and on the long term during wound recovery (Lowell 1991; Toth and Pavia 2006). Moreover, we measured only young thalli and the range in stipe cross-sectional area was much smaller than when both young and mature thalli were sampled. Including mature and thus larger thalli in the analysis may yield a significant correlation between break force and stipe cross-sectional area.

Apparently at this size range, the risk of dislodgement due to drag as determined by thallus planform area does not (yet) dictate size of attachment and thallus. Measuring a larger range of thallus sizes, and thus higher expected drag, may produce a significant effect (cf. discussion by Thomsen and Wernberg 2005). Additionally, drag will not only increase due to growth, but also with degree of branching (Starko et al. 2014), and may therefore become an even more decisive selective pressure for older, higher branched, thalli. Our observations support the expectation that for larger, mature thalli break force will start to scale with thallus size. If we include data on one very large thallus, the correlations between thallus size and break force become significant, and when considering only large thalli (>ca. 5000 mm² planform area, Fig. 6), data indicates a linear trend between thallus area and break force.

Medium and large thalli were less common at the dock site compared to the surrounding rocks. The observed size distribution at the rocks is a stable and valid reference, as we found a very comparable distribution to Haring et al. (2002), who sampled in the area almost 20 years earlier and found ≈70% small, ≈30% medium, and <5% large thalli, despite a different sampling method (values extracted from Fig. 7). Apparently, there is a limitation in thallus size that is specific to the dock surface. If thalli there continue to grow larger without being attached stronger, and thus experience more drag due to their increased size, then they have a higher risk for dislodgement. The highly heterogeneous and weak substrate could limit disc growth, as competition with other organisms for suitable attachment space on the densely colonized rough and sediment-filled surface may be severe. And even if discs do grow larger and start to scale with thallus size for mature thalli (see discussion in previous paragraph), the substrate may impose a sort of “absolute” limit to adhesion, as larger discs may encompass so much weak or loose material that they do not adhere stronger. As discussed above, substrate failure was commonly observed in dislodgement tests. Spots with strong, cohesive substrate for “good” adhesion, such as clean pebbles in the concrete, are rare, so only very few thalli can grow large without being dislodged. This indicates that thallus size may be mechanically limited at the dock site. Even though the sampling sites dock and rock were spatially very close and the substrate the most stringent difference, sites may also differ in other aspect such as small scale flow regime, light regime or herbivore pressure, which could affect life stages differently and thereby result in a different size distribution.

As an intertidal species, *F. distichus* does not live permanently submerged, but regularly surfaces at low tides. At the sampled sites, summer low tides fall during midday and the thalli may desiccate strongly. Desiccation renders the stipes of *F. distichus* more brittle, they require less total energy to break, and easily break when bent repeatedly (Haring et al. 2002). Mortality may thus not only arise from drag forces exceeding the attachment’s resistance, but also (or even primarily) from relatively small waves that hit the thalli when they are desiccated.

The attachment disc of *F. distichus* is a comparable simple attachment that anchors the entire thallus to a single spot, and, in comparison to soil-penetrating roots of land plants, provides superficial adhesion to hard-bottom substrates. However, land plants too have evolved forms with superficial attachment. Climbing plants attach to other plants or inanimate structures in order to grow upward toward the light (e.g., Darwin 1882). Some climbing plants, such as Boston ivy (*Parthenocissus tricuspidata*) or the passion flower *Passiflora discophora*, use specialized coiled and branched tendrils equipped with multiple attachment discs, which allow them to anchor to vertical surfaces (Steinbrecher et al. 2011; Bohn et al. 2015). Mechanical features of these flat and superficial attachments include energy dissipation via sequential failure of several attachment points, energy storing springs, and the redundancy of multiple attachment points (Steinbrecher et al. 2010; Klimm et al. 2022, 2024). Similarly, more complex marine attachments than individual discs (Fig. 1B, C) may possess additional biomechanical features yet to be identified.

## Conclusion

*Fucus distichus* attaches via a disc that provides tight form closure to the heterogeneous test substrate. The connection from disc to blades is assured by a weak and compliant but extensible and thick stipe. The disc attachment is weaker than the connecting stipe, probably due to weak substrate cohesion, but due to the large contact area, failure location within the attachment is balanced. This highlights the importance of the stipe broadening into a disc for attachment. Continuous measurements of force and displacement during dislodgement show the work required to dislodge the attachment is mainly required to stretch the stipes in their elastic range up to failure. Thalli bent and streamlined at water velocities far below peak values, which will reduce the load the attachment experiences. Our measurements confirm general trends in seaweed biomechanics; namely the scaling of break force with thallus size across populations, thick but weak stipes as a typical feature for brown algae compared to red algae, low strength and stiffness but high extensibility compared to other biomaterials, and high flexibility allowing passive streamlining in water flow. Unlike our expectation, the attachment did not scale with thallus size for the young thalli included in testing, indicating that the risk of dislodgement due to drag does not (yet) dictate thallus size for young thalli. Future studies should include a larger size range of thalli to investigate this relation. Our results do show that there exist upper limits in thallus size at the sampled site compared to the surrounding rocks, and suggest that these are mechanical limits which result from the weak substrate. The attachment disc of *F. distichus* represents a comparable simple attachment structure, and detailed analyses of more complex structures may provide additional functional features of the attachment of marine macroalgae.

## Supplementary Material

obag010_Supplemental_File

## Data Availability

The data underlying this article will be shared on reasonable request to the corresponding author.

## References

[bib1] Amsler CD, Neushul M. 1989. Chemotactic effects of nutrients on spores of the kelps *Macrocytis pyrifera* and *Pterygophora california*. Mar Biol 102(4):557–64. 10.1007/BF00438358

[bib2] Ang PO . 1991a. Natural dynamics of a *Fucus distichus* (Phaeophyceae, Fucales) population: reproduction and recruitment. Mar Ecol Prog Ser 78:71–85. 10.3354/meps078071

[bib3] Ang PO . 1991b. Age- and size-dependent growth and mortality in a population of *Fucus distichus*. Mar Ecol Prog Ser 78:173–87. 10.3354/meps078173

[bib4] Barnes H, Topinka JA. 1969. Effect of the nature of the substratum on the force required to detach a common littoral alga. Am Zool 9(3):753–8. 10.1093/icb/9.3.753

[bib5] Bauer U, Poppinga S, Müller UK. 2020. Mechanical ecology—taking biomechanics to the field. Integr Comp Biol 60(4):820–8. 10.1093/icb/icaa018 32275745

[bib6] Bohn HF, Günther F, Fink S, Speck T. 2015. A passionate free climber: structural development and functional morphology of the adhesive tendrils in *Passiflora discophora*. Int J Plant Sci 176(3):294–305. 10.1086/680231

[bib7] Burnett NP, Gaylord B. 2022. Flow, form, and force: methods and frameworks for field studies of macroalgal biomechanics. J Exp Bot 73(4):1122–38. 10.1093/jxb/erab498 34791153

[bib8] Burnett NP, Koehl MAR. 2019. Mechanical properties of the wave-swept kelp *Egregia menziesii* change with season, growth rate and herbivore wounds. J Exp Biol 222(4):jeb190595. 10.1242/jeb.190595 30679240

[bib9] Burnett NP, Koehl MAR. 2021. Age affects the strain-rate dependence of mechanical properties of kelp tissues. Am J Bot 108(5):769–76. 10.1002/ajb2.1662 33993474

[bib10] Burnett NP, Koehl MAR. 2022. Ecological biomechanics of damage to macroalgae. Front Plant Sci 13:981904. 10.3389/fpls.2022.981904 36092422 PMC9452655

[bib11] Carrington E . 1990. Drag and dislodgement of an intertidal macroalga: consequences of morphological variation *in Mastocarpus papillatus* Kützing. J Exp Mar Biol Ecol 139(3):185–200. 10.1016/0022-0981(90)90146-4

[bib12] Cohen J . 1988. Statistical power analysis for the behavioral sciences. 2nd ed. USA: Lawrence Erlbaum Associates. 10.4324/9780203771587

[bib13] Darwin C . 1882. The movements and habits of climbing plants. London: John Murray.

[bib14] Daweson EY . 1966. Marine botany: An introduction. New York, NY: Holt, Rinehart and Winston, Inc.

[bib15] De Bettignies T, Wernberg T, Lavery PS. 2013. Size, not morphology, determines hydrodynamic performance of a kelp during peak flow. Mar Biol 160(4):843–51. 10.1007/s00227-012-2138-8

[bib16] Demes KW, Starko S, Harley CD. 2020. Multiple stressors drive convergent evolution of performance properties in marine macrophytes. New Phytol 229(4):2311–23. 10.1111/nph.16994 33037641

[bib17] Denny MW, Gaylord B. 2002. The mechanics of wave-swept algae. J Exp Biol 205(10):1355–62. 10.1242/jeb.205.10.1355 11976348

[bib18] Druehl L, Clarkston B. 2016. Pacific seaweeds. A guide to common seaweeds of the west coast. Madeira Park: Harbour Publishing.

[bib19] Dudgeon SR, Johnson AS. 1992. Thick vs. thin: thallus morphology and tissue mechanics influence differential drag and dislodgement of two co-dominant seaweeds. J Exp Mar Biol Ecol 165:23–43. 10.1016/0022-0981(92)90287-K

[bib20] Friedland MT, Denny MW. 1995. Surviving hydrodynamic forces in a wave-swept environment: consequences of morphology in the feather boa kelp, *Egregia menziesii* (Turner). J Exp Mar Biol Ecol 190(1):109–33. 10.1016/0022-0981(95)00038-s

[bib21] Gaylord B . 2000. Biological implications of surf-zone flow complexity. Limnol Oceanogr 45(1):174–88. 10.4319/lo.2000.45.1.0174

[bib22] Gaylord B, Denny MW, Koehl MAR. 2008. Flow forces on seaweeds: field evidence for roles of wave impingement and organism inertia. Biol Bull 215(3):295–308. 10.2307/25470713 19098150

[bib23] Harder DL, Hurd CL, Speck T. 2006. Comparison of mechanical properties of four large, wave-exposed seaweeds. Am J Bot 93:1426–32. 10.3732/ajb.93.10.1426 21642089

[bib24] Harder DL, Speck O, Hurd CL, Speck T. 2004. Reconfiguration as a prerequisite for survival in highly unstable flow-dominated habitats. J Plant Growth Regul 23:98–107. 10.1007/s00344-004-0043-1

[bib25] Hardy FG, Moss BL. 1979. The effects of the substratum on the morphology of the rhizoids of *Fucus* germlings. Estuar Coast Mar Sci 9(5):577–84. 10.1016/0302-3524(79)90081-1

[bib26] Haring RN, Carpenter RC. 2007. Habitat-induced morphological variation influences photosynthesis and drag on the marine macroalga *Pachydictyon coriaceum*. Mar Biol 151(1):243–55. 10.1007/s00227-006-0474-2

[bib27] Haring RN, Dethier MN, Williams SL. 2002. Desiccation facilitates wave-induced mortality of the intertidal alga *Fucus gardneri*. Mar Ecol Prog Ser 232:75–82. 10.3354/meps232075

[bib28] Hatchett WJ, Coyer JA, Sjøtun K, Jueterbock A, Hoarau G. 2022. A review of reproduction in the seaweed genus *Fucus* (Ochrophyta, Fucales): background for renewed consideration as a model organism. Front Mar Sci 9:1051838. 10.3389/fmars.2022.1051838

[bib29] Holbrook NM, Denny MW, Koehl MAR. 1991. Intertidal ‘trees’: consequences of aggregation on the mechanical and photosynthetic properties of sea-palms *Postelsia palmaeformis* Ruprecht. J Exp Mar Biol Ecol 146:39–67. 10.1016/0022-0981(91)90254-T

[bib30] Jonsson PR, Granhag L, Moschella PS, Åberg P, Hawkins SJ, Thompson RC. 2006. Interactions between wave action and grazing control the distribution of intertidal macroalgae. Ecology 87(5):1169–78. 10.1890/0012-9658(2006)87[1169:ibwaag]2.0.co;2 16761596

[bib31] Kiirikki M, Ruuskanen A. 2009. How does *Fucus vesiculosus* survive ice scraping? Bot Mar 39:133–40. 10.1515/botm.1996.39.1-6.133

[bib32] Klimm F, Schmier S, Bohn HF, Kleiser S, Thielen M, Speck T. 2022. Biomechanics of tendrils and adhesive pads of the climbing passion flower *Passiflora discophora*. J Exp Bot 73(4):1190–203. 10.1093/jxb/erab456 34673926 PMC8866636

[bib33] Klimm F, Thielen M, Homburger J, Modert M, Speck T. 2024. Natural coil springs: biomechanics and morphology of the coiled tendrils of the climbing passion flower *Passiflora discophora*. Acta Biomater 189:478–90. 10.1016/j.actbio.2024.10.002 39393657

[bib34] Koehl MAR . 1979. Stiffness or extensibility of intertidal algae: a comparative study of modes of withstanding wave action. J Biomech 12(8):634. 10.1016/0021-9290(79)90128-3

[bib35] Koehl MAR . 1984. How do benthic organisms withstand moving water? Am Zool 24:57–70. 10.1093/icb/24.1.57

[bib36] Koehl MAR . 1986. Seaweeds in moving water: form and mechanical function. In: Givnish T. J., editor. On the economy of plant form and function. New York, NY: Cambridge University Press. p. 603–34.

[bib37] Koehl MAR . 2000. Mechanical design and hydrodynamics of blade-like algae: chondracanthus exasperatus. In: Spatz H. C., Speck T. eds., Proceedings of the Third International Plant Biomechanics Conference. Thieme Verlag, Stuttgart. p. 295–308.

[bib38] Koehl MAR . 2022. Ecological biomechanics of marine macrophytes. J Exp Bot 73(4):1104–21. 10.1093/jxb/erab536 35199170

[bib39] Koehl MAR, Wainwright SA. 1977. Mechanical adaptations of a giant kelp. Limnol Oceanogr 22(6):1067–71. 10.4319/lo.1977.22.6.1067

[bib40] Krumhansl KA, Demes KW, Carrington E, Harley CD. 2015. Divergent growth strategies between red algae and kelps influence biomechanical properties. Am J Bot 102(11):1938–44. 10.3732/ajb.1500289 26546127

[bib41] Lowell RB, Markham JH, Mann KH. 1991. Herbivore-like damage induces increased strength and toughness in a seaweed. Proc R Soc Lond B 243(1306):31–8. 10.1098/rspb.1991.0006

[bib42] Malm T, Engkvist R, Kautsky L. 1999. Grazing effects of two freshwater snails on juvenile *Fucus vesiculosus* in the Baltic Sea. Mar Ecol Prog Ser 188: 63–71. 10.3354/meps188063

[bib43] Malm T, Kautsky L, Claesson T. 2003. The density and survival of *Fucus vesiculosus* L. (Fucales, Phaeophyta) on different bedrock types on a Baltic Sea moraine coast. Bot Mar 46(3):256–62. 10.1515/BOT.2003.023

[bib44] Martone PT . 2007. Kelp versus coralline: cellular basis for mechanical strength in the wave-swept seaweed *Calliarthron* (Corallinaceae, Rhodophyta). J Phycol 43(5):882–91. 10.1111/j.1529-8817.2007.00397.x

[bib45] Martone PT, Kost L, Boller M. 2012. Drag reduction in wave-swept macroalgae: alternative strategies and new predictions. Am J Bot 99(5):806–15. 10.3732/ajb.1100541 22523350

[bib46] McCook LJ, Chapman ARO. 1992. Vegetative regeneration of *Fucus* rockweed canopy as a mechanism of secondary succession on an exposed rocky shore. Botan Mar 35(1):35–46. 10.1515/botm.1992.35.1.35

[bib47] Morrison L, Feely M, Stengel DB, Blamey N, Dockery P, Sherlock A, Timmins É. 2009. Seaweed attachment to bedrock: biophysical evidence for a new geophycology paradigm. Geobiology 7(4):477–87. 10.1111/j.1472-4669.2009.00206.x 19624752

[bib48] Moss B . 1948. Studies in the genus *Fucus*: III. Structure and development of the attaching discs of Fucus vesiculosus. Ann Bot 14(55):411–9. 10.1093/oxfordjournals.aob.a083255

[bib49] Moss B . 1967. The apical meristem of *Fucus*. New Phytol 66(1):67–74. 10.1111/j.1469-8137.1967.tb05988.x

[bib50] Niklas KJ . 1992. Plant biomechanics. An engineering approach to plant form and function. Chicago, IL: The University of Chicago Press.

[bib51] Niklas KJ . 1995. Plant height and the properties of some herbaceaous stems. Ann Bot 75:133–42. 10.1006/anbo.1995.1004

[bib52] Powell HT . 1957. Studies in the genus *Fucus* L. II. Distribution and ecology of forms of *Fucus distichus* L. Emend. Powell in Britain and Ireland. J Mar Biol Ass 36:663–93. 10.1017/S0025315400025923

[bib53] R Core Team. 2019. R: a language and environment for statistical computing. Vienna: R Foundation for Statistical Computing.

[bib54] Schiel DR, Foster MS. 2006. The population biology of large brown seaweeds: ecological consequences of multiphase life histories in dynamic coastal environments. Annu Rev Ecol Evol Syst 37:343–72. 10.1146/annurev.ecolsys.37.091305.110251

[bib55] Starko S, Claman BZ, Martone PT. 2014. Biomechanical consequences of branching in flexible wave-swept macroalgae. New Phytol 206(1):133–40. 10.1111/nph.13182 25413976

[bib56] Starko S, Martone PT. 2016. Evidence of an evolutionary-developmental trade-off between drag avoidance and tolerance strategies in wave-swept intertidal kelps (Laminariales, Phaeophyceae). J Phycol 52:54–63. 10.1111/jpy.12368 26987088

[bib57] Steinbrecher T, Beuchle G, Melzer B, Speck T, Kraft O, Schwaiger R. 2011. Structural development and morphology of the attachment system of *Parthenocissus tricuspidata*. Int J Plant Sci 172(9):1120–9. 10.1086/662129

[bib58] Steinbrecher T, Danninger E, Harder D, Speck T, Kraft O, Schwaiger R. 2010. Quantifying the attachment strength of climbing plants: a new approach. Acta Biomater 6(4):1497–504. 10.1016/j.actbio.2009.10.003 19818882

[bib59] Stevens CL, Hurd CL, Smith MJ. 2002. Field measurement of the dynamics of the bull kelp *Durvillaea antarctica* (Chamisso) Heriot. J Exp Mar Biol Ecol 269(2):147–71. 10.1016/S0022-0981(02)00007-2

[bib60] Tarakhovskaya ER . 2014. Mechanisms of bioadhesion of macrophytic algae. Russ J Plant Physiol 61(1):19–25. 10.1134/S1021443714010154

[bib61] Thomsen MS . 2004. Species, thallus size and substrate determine macroalgal break force and break location in a low-energy soft-bottom lagoon. Aquat Bot 80(2):153–61. 10.1016/j.aquabot.2004.08.002

[bib62] Thomsen MS, Wernberg T. 2005. Miniview: what affects the forces required to break or dislodge macroalgae? Eur J Phycol 40(2):139–48. 10.1080/09670260500123591

[bib63] Toth GB, Pavia H. 2006. Artificial wounding decreases plant biomass and shoot strength of the brown seaweed *Ascophyllum nodosum* (Fucales, Phaeophyceae). Mar Biol 148(6):1193–9. https://doi.org/10.100\7/s00227-005-0167-2

[bib64] van Tamelen PG, Stekoll MS. 1997. The role of barnacles in the recruitment and subsequent survival of the brown alga, *Fucus gardneri* (Silva). J Exp Mar Biol Ecol 208:227–38. 10.1016/S0022-0981(96)02664-0

[bib65] Vogel S . 1994. Life in moving fluids: the physical biology of flow. Boston, MA: Willard Grant Press. p. 70.

[bib66] Wikström SA, von Wachenfeldt T, Kautsky L. 2002. Establishment of the exotic species *Fucus evanescens* C. Ag. (Phaeophyceae) in Öresund, Southern Sweden. Bot Mar 45(6):510–7. 10.1515/BOT.2002.054

