# Supplementary material for: Biomechanics of the Attachment of the Intertidal Seaweed Fucus distichus
Source: Integr Org Biol. 2026 Mar 27;8(1):obag010. doi: 10.1093/iob/obag010 (PMC13080366; doi:10.1093/iob/obag010)
Supplement: obag010_Supplemental_File [file obag010_supplemental_file.pdf]

Supplementary material

Biomechanics of the attachment of the intertidal seaweed *Fucus distichus*

Frederike Klimm[1,2], Adam P. Summers[3], Thomas Speck[1,2,*]

[1]Plant Biomechanics Group @ Botanic Garden, University of Freiburg, Freiburg, Germany

[2]Cluster of Excellence livMatS @ FIT – Freiburg Center for Interactive Materials and Bioinspired Technologies, University of Freiburg, Germany

[3]Friday Harbor Laboratories, University of Washington, USA

*Corresponding author:

Thomas Speck
Botanic Garden, University of Freiburg
Schänzlestraße 1
D-79104 Freiburg, Germany
thomas.speck@biologie.uni-freiburg.de

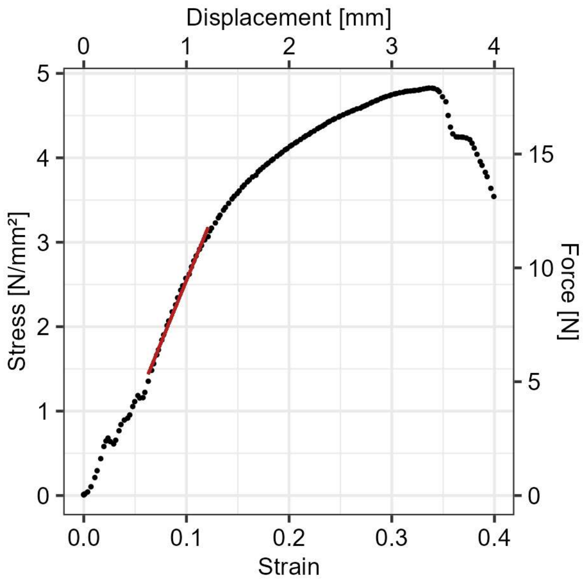

Fig. S1: Determination of elastic modulus from stipe extension data. An example stress-strain curve illustrates the selection of a linear region after initial artefacts and prior to potential onset of slipping. The elastic modulus *E* was calculated as slope of the linear regression line (red).

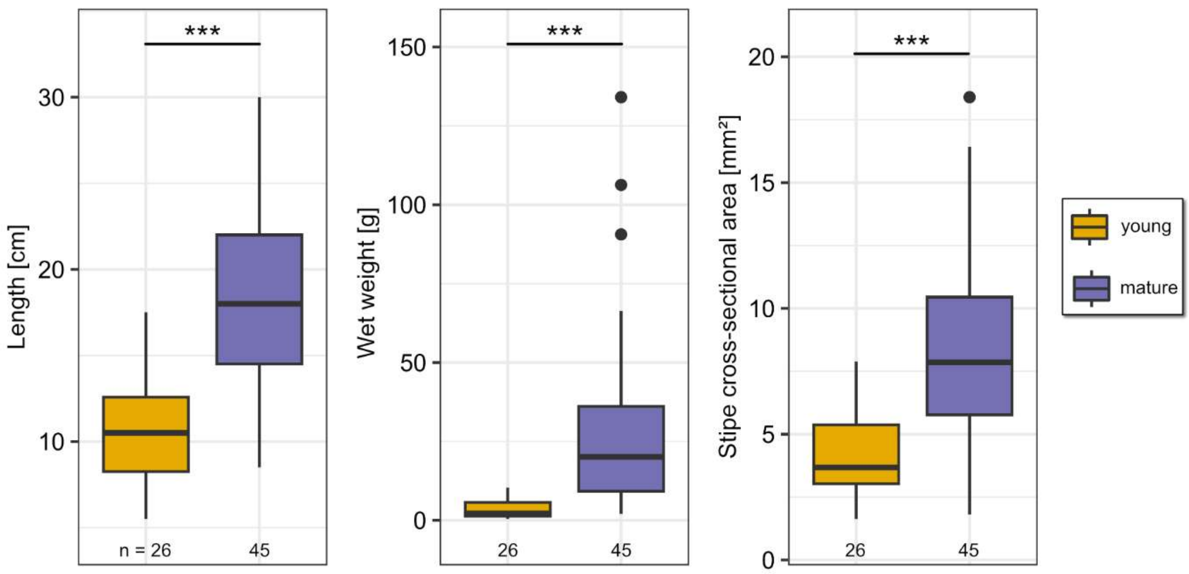

Fig. S2: Thallus morphology for samples tested in tensile tests. Thallus length, thallus wet weight and stipe cross-sectional area of *F. distichus* are plotted. All three properties differed significantly between young and mature thalli (Wilcoxon test, $p = 2 \times 10^{-8}$, $p = 3 \times 10^{-9}$, $p = 1 \times 10^{-8}$).

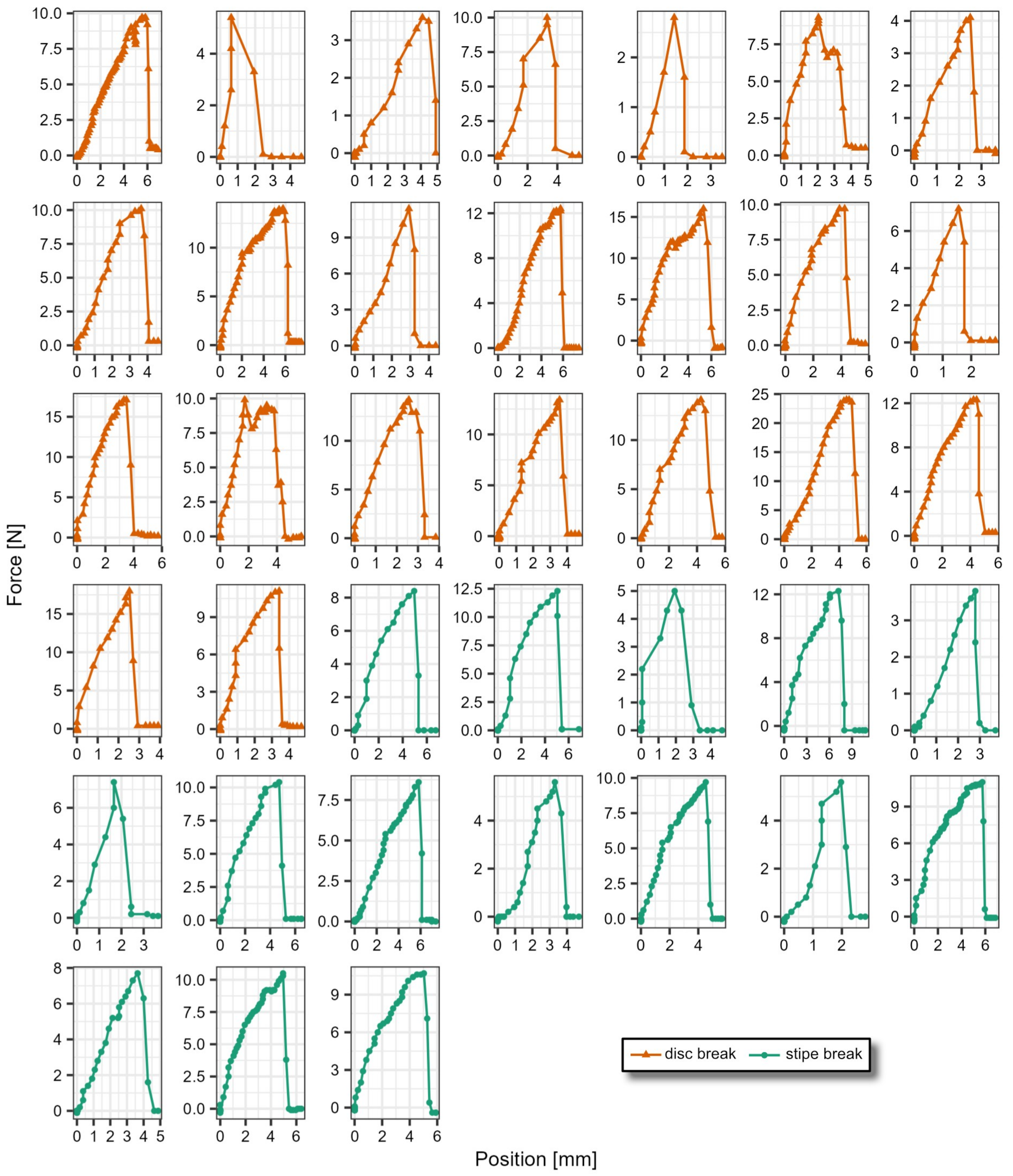

Fig. S3: Dislodgement of *F. distichus*. Force and displacement are plotted for young thalli dislodged in the field at their attachment (disc and lower stipe). Samples that failed at the stipe are plotted in orange (triangles), and samples that failed at their disc in green (circles).

Tab. S1: Statistical data for correlations between break force and morphological variables. Abbreviations are n: sample size; r: Pearson's correlation coefficient; p: p-value; p_{bonf}: Bonferroni corrected p-value; des: detectable effect size (correlation coefficient that could be detected with test power 0.8 for given sample size and alpha level 0.05); and des_{bonf} detectable effect size for Bonferroni corrected alpha level.

Variable	Full dataset								Dataset without extreme value				
	n	r	p		des	p_{bonf}		des_{bonf}	r	p		p_{bonf}	
Stipe cross-sect. area	38	0.56	0.000288	***	0.44	2E-03	**	0.53	0.42	9E-03	**	6E-02	n.s.
Disc area	29	0.77	1.18E-06	***	0.49	7E-06	***	0.59	0.68	6E-05	***	4E-04	***
Disc wet weight	30	0.60	0.000445	***	0.49	3E-03	**	0.58	0.54	3E-03	**	2E-02	*
Thallus length	38	0.45	0.004746	**	0.44	3E-02	*	0.53	0.22	2E-01	n.s.	1	n.s.
Thallus area	37	0.49	0.002003	**	0.44	1E-02	*	0.53	0.25	1E-01	n.s.	8E-01	n.s.
Thallus wet weight	38	0.46	0.003609	**	0.44	2E-02	*	0.53	0.22	2E-01	n.s.	1	n.s.

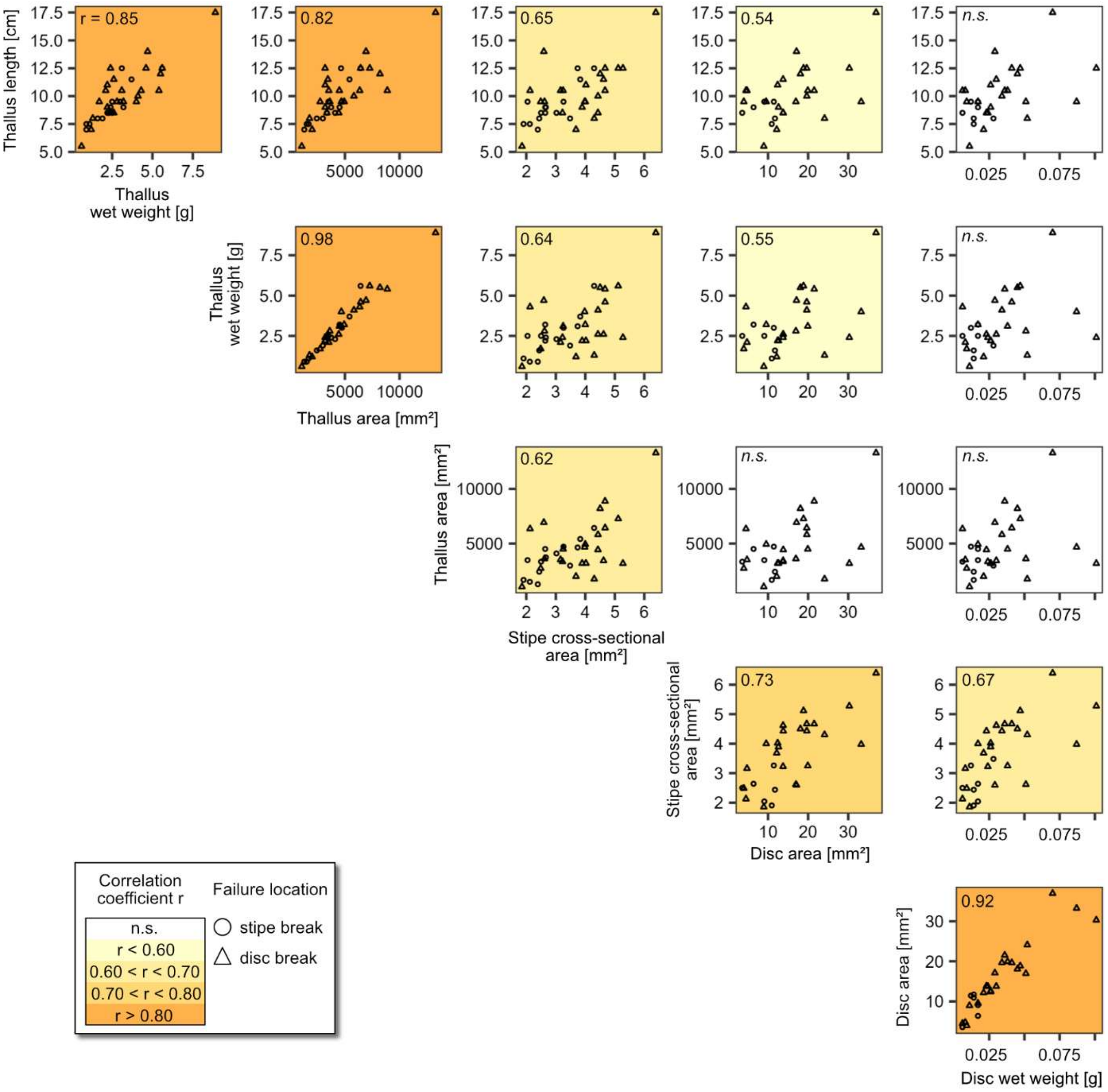

Fig. S4: Thallus and attachment sizing of *F. distichus* dislodged in the field. Correlations between between thallus length, thallus wet weight, thallus planform area, stipe cross-sectional area, disc area and disc wet weight are plotted, for *F. distichus* that failed at the stipe (circles) and the disc (triangles). Values for Pearson's correlation coefficient r are color coded in different shades of orange or remain uncolored if n.s. after Bonferroni-correction. See table S2 for detailed values

Tab. S2: Statistical data for correlation matrix of morphology variables. Abbreviations are n: sample size; r: Pearson's correlation coefficient; p: p-value; p_{bonf}: Bonferroni-corrected p-value; des: detectable effect size (correlation coefficient that could be detected with test power 0.8 for given sample size and alpha level 0.05); and des_{bonf} detectable effect size for Bonferroni-corrected alpha level.

Variables		Full dataset								Dataset without extreme value				
		n	r	p		des	p_{bonf}		des_{bonf}	r	p		p_{bonf}	
Thallus length	Thallus area	37	0.82	4E-10	***	0.44	7E-09	***	0.57	0.73	4E-07	***	6E-06	***
Thallus length	Thallus wet weight	38	0.85	2E-11	***	0.44	4E-10	***	0.56	0.77	2E-08	***	3E-07	***
Thallus area	Thallus wet weight	37	0.98	4E-26	***	0.44	6E-25	***	0.57	0.97	2E-22	***	2E-21	***
Stipe cross-sect. area	Disc area	29	0.73	8E-06	***	0.49	1E-04	***	0.63	0.65	2E-04	***	3E-03	**
Stipe cross-sect. area	Disc wet weight	30	0.67	6E-05	***	0.49	9E-04	***	0.62	0.62	3E-04	***	5E-03	**
Disc area	Disc wet weight	29	0.92	2E-12	***	0.49	3E-11	***	0.63	0.93	1E-12	***	2E-11	***
Thallus length	Stipe cross-sect. area	38	0.65	8E-06	***	0.44	1E-04	***	0.56	0.55	5E-04	***	7E-03	**
Thallus length	Disc area	29	0.54	2E-03	**	0.49	4E-02	*	0.63	0.35	7E-02	n.s.	1	n.s.
Thallus length	Disc wet weight	30	0.49	6E-03	**	0.49	9E-02	n.s.	0.62	0.4	3E-02	*	5E-01	n.s.
Thallus area	Stipe cross-sect. area	37	0.62	4E-05	***	0.44	6E-04	***	0.57	0.49	2E-03	**	4E-02	*
Thallus area	Disc area	29	0.5	6E-03	**	0.49	9E-02	n.s.	0.63	0.26	2E-01	n.s.	1	n.s.
Thallus area	Disc wet weight	30	0.34	7E-02	n.s.	0.49	1E+00	n.s.	0.62	0.18	3E-01	n.s.	1	n.s.
Thallus wet weight	Stipe cross-sect. area	38	0.64	1E-05	***	0.44	2E-04	***	0.56	0.52	9E-04	***	1E-02	*
Thallus wet weight	Disc area	29	0.55	2E-03	**	0.49	3E-02	*	0.63	0.35	7E-02	n.s.	1	n.s.
Thallus wet weight	Disc wet weight	30	0.41	2E-02	*	0.49	3E-01	n.s.	0.62	0.29	1E-01	n.s.	1	n.s.
